# Structural basis of bacteriophage T5 infection trigger and *E. coli* cell wall perforation

**DOI:** 10.1126/sciadv.ade9674

**Published:** 2023-03-24

**Authors:** Romain Linares, Charles-Adrien Arnaud, Grégory Effantin, Claudine Darnault, Nathan Hugo Epalle, Elisabetta Boeri Erba, Guy Schoehn, Cécile Breyton

**Affiliations:** Univ. Grenoble Alpes, CNRS, CEA, IBS, Grenoble F-38000, France.

## Abstract

Most bacteriophages present a tail allowing host recognition, cell wall perforation, and viral DNA channeling from the capsid to the infected bacterium cytoplasm. The majority of tailed phages bear a long flexible tail (*Siphoviridae*) at the tip of which receptor binding proteins (RBPs) specifically interact with their host, triggering infection. In siphophage T5, the unique RBP is located at the extremity of a central fiber. We present the structures of T5 tail tip, determined by cryo–electron microscopy before and after interaction with its *E. coli* receptor, FhuA, reconstituted into nanodisc. These structures bring out the important conformational changes undergone by T5 tail tip upon infection, which include bending of T5 central fiber on the side of the tail tip, tail anchoring to the membrane, tail tube opening, and formation of a transmembrane channel. The data allow to detail the first steps of an otherwise undescribed infection mechanism.

## Teaser

Structure of a siphophage tail tip interacting with its nanodisc-solubilised receptor reveals a novel viral entry mechanism.

## INTRODUCTION

Bacteriophages or phages, viruses that infect bacteria, represent the most abundant biological entity on our planet. They are present in all ecosystems where bacteria develop and outnumber their hosts by at least an order of magnitude, being instrumental in the development and evolution of microbial populations (*1*). Moreover, with the increasing number of pathogenic strains resistant to antibiotics, virulent phages are considered as a serious alternative or complement to classical treatments (*2*). The vast majority of known phages bear a tail whose tip serves to recognize the host, perforate the bacterial cell wall, and deliver the viral genome into the host cytoplasm. Tails can be long and contractile in *Myoviridae*, long and flexible in *Siphoviridae*, or short in *Podoviridae*. Bacterial tail–like machines also serve as a means to inject various macromolecules in neighboring prokaryotic and/or eukaryotic cells: All these systems derive from a common ancestor that share high structural similarities (*3*–*6*). The contracting tails and bacterial tail–like systems have seen their mechanism of cell wall perforation described in exquisite details: They literally drill a hole through the cell wall by contracting an outer sheath that propels a needled inner tube in a syringe-like manner (*5*, *7*–*9*). On the contrary, very little is known for siphophages, which represent more than 60% of all phages (*10*). Structures of the tail tube before and after interaction with the receptor suggest that the tail tube does not play a direct role in infection (*11*, *12*). Structures of isolated purified baseplate (*13*–*15*) and of a tail tip (*16*) of Gram-positive infecting siphophages are available, and lactophage p2 purified baseplate was solved in a closed and open state, suggesting activation for this sugar-binding siphophage (*15*). However, the mechanism of transmission of host recognition, tail tube opening, and cell wall perforation remains completely unknown in siphophages.

**Fig. 1.**
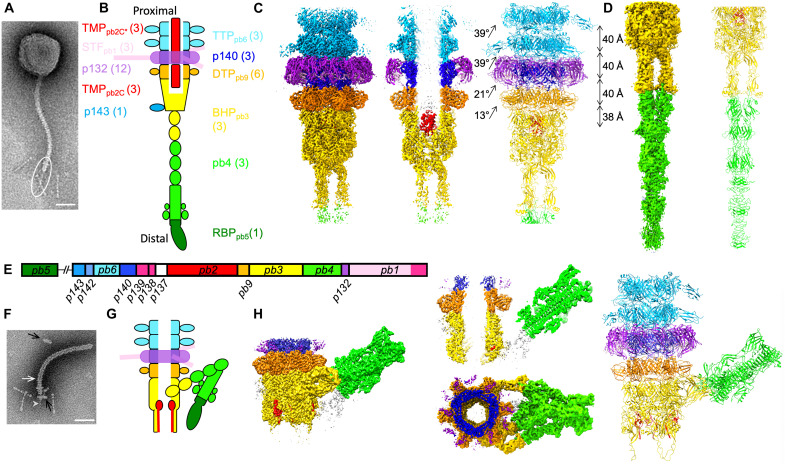
Structure of T5 tail tip before and after interaction with FhuA in nanodisc. (**A**) Negative stain EM image of phage T5, with the tail tip circled. (**B**) Scheme of T5 tail tip with the assignment of the different proteins and their copy number. (**C**) Cryo-EM structure of T5 upper tail tip at 3.5-Å resolution. Left: Isosurface view of the map seen from the side; middle: central slice side view; right: ribbon representation of the modeled proteins. The twist and rise between each ring are noted. (**D**) Cryo-EM structure of the central fiber at 4.2-Å resolution. Left: Isosurface view of the map seen from the side; right: ribbon representation of the modeled proteins. (**E**) Map of T5 tail structural proteins and genes. (**F**) Negative stain EM image of a T5 tail interacting with a FhuA nanodisc (black arrows). The white arrow points to the empty-filled limit of the tail (see also extended data Fig. 1F). The gray arrowhead points to a density going through the nanodisc. (**G**) Scheme of T5 tip after interaction with FhuA (Tip-FhuA). (**H**) Cryo-EM structure of Tip-FhuA at resolutions ranging from 3.6- to 4.3-Å resolution. Isosurface view of a Tip-FhuA composite map seen from the side (left), a central slice side view of it (middle top), and a top view (middle bottom). This composite map is formed by the addition of Tip-FhuA C3 open tube and C1 bent fiber maps and is only for visualization purposes; right: ribbon representation of the modeled proteins. The color code in (C), (D), (G), and (H) is the same as in (B). Unattributed densities are in white. Scale bars, 50 nm.

Phage T5 (*17*), a *Siphoviridae* infecting *Escherichia coli*, is a model phage belonging to the T series introduced by Delbrück and co-workers in the 1940s (*18*). It presents a 90-nm icosahedral capsid (*19*) to which is attached a 160-nm tail tube (Fig. 1A), formed by the polymerization of 40 ring-shaped trimers of the tail tube protein pb6 (TTP_pb6_) (*11*) around the tape measure protein pb2 (TMP_pb2_) (*20*). At its distal end, the tail harbors the tip complex, also called baseplate: three dispensable L-shaped side tail fibers (LTF_pb1_) reversibly bind to the sugar moiety of the host lipopolysaccharide (*21*). They are linked, by the collar, to a conical structure formed by the distal tail protein pb9 (DTP_pb9_) (*22*) and the baseplate hub protein pb3 (BHP_pb3_, also called tail-associated lysin/lysozyme) (*17*). A central fiber protein (pb4), at the extremity of which is found the receptor binding protein pb5 (RBP_pb5_) (*23*, *24*), completes the tip complex, with p140 and p132 of unknown location (Fig. 1B) (*17*). FhuA, an outer membrane *E. coli* iron-ferrichrome transporter, is the bacterial receptor recognized by T5 (*25*). The mere interaction of T5 with its purified receptor FhuA triggers the release of viral DNA in vitro (*26*), making this phage an excellent model for studying host recognition, DNA ejection (*11*, *27*), and cell wall perforation mechanisms. We thus embarked on solving the structure of T5 tail tip before (Tip) and after (Tip-FhuA) interaction with its receptor; our results unravel the conformational changes underwent by T5 tail tip and allow to detail the molecular mechanism of tail opening and outer membrane perforation.

## RESULTS

### General architecture

T5 tails (*11*, *17*) were preferred over whole phages for cryo–electron microscopy (cryo-EM) as they allow better quality imaging (fig. S1A). Purified tails, obtained from a mutant bearing an amber mutation in the major capsid protein, behave as capsid-attached tails: They interact with their receptor and perforate outer membrane vesicles (*17*). Micrograph acquisition and extensive image processing (fig. S2 and table S1) yielded three maps of different tip subcomponents (Fig. 1, C and D, and fig. S1C), whose resolution allowed tracing all the proteins from the tail tube to the distal end of the central fiber, except for LTF_pb1_ and RBP_pb5_ (table S1). After two TTP_pb6_ trimeric rings, the tube continues with a p140 trimer and then a DTP_pb9_ hexamer. A BHP_pb3_ trimer closes the tube and forms the beginning of the central fiber that continues with a pb4 trimer. The p140 trimeric ring is surrounded by a p132 dodecamer that forms the collar, onto which are grafted three LTF_pb1_.

Upon T5 tail incubation with detergent-solubilized FhuA, BHP_pb3_ opens, TMP_pb2_ is expelled from the tube lumen, and the central fiber disappears (fig. S1F, inset) (*11*). As the presence of a lipid bilayer might stabilize a cell wall perforation intermediate, we used instead FhuA reconstituted into nanodiscs. Nanodiscs are little patches of lipid bilayers stabilized by a membrane scaffold protein (MSP) (*28*). We used the largest MSP available to provide a lipid bilayer in addition to reconstituted FhuA (see also Material and Methods). Images of FhuA nanodisc–incubated tails clearly show (i) the presence of a nanodisc perpendicular to the tail tube at the rim of the open BHP_pb3_, (ii) a protrusion going through the nanodisc, (iii) TMP_pb2_ partial ejection from the tail tube lumen, and (iv) the bending of the central fiber with a very acute angle on one side of the tip (Fig. 1F and fig. S1F), suggesting that we trapped an ejection intermediate. Extensive cryo-EM image processing yielded three other maps (Fig. 1H, figs. S1H and S2, and table S1) allowing tracing all T5 tip proteins, except, again, LTF_pb1_ and RBP_pb5_. Densities belonging to RBP_pb5_ are visible, but resolution is insufficient to build a model. However, a small-angle neutron scattering envelop of the FhuA-RBP_pb5_ complex (*24*) and our recently determined FhuA-RBP_pb5_ structure (*29*) could be very well fitted into the densities (fig. S3D).

**Fig. 2.**
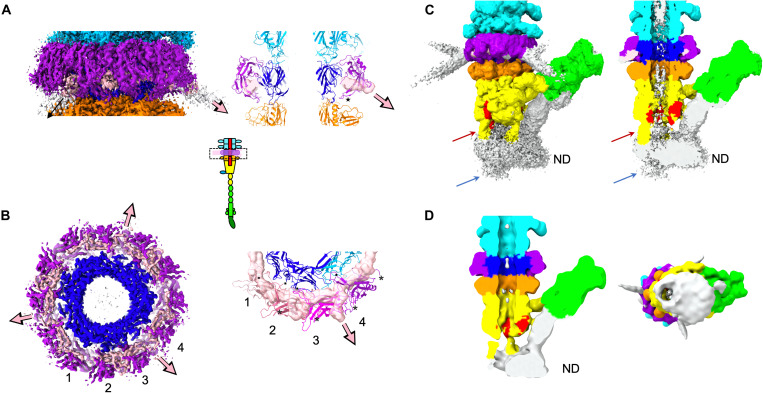
Structure of T5 Tip collar and Tip-FhuA complex. (**A**) Left: Isosurface side view of the tip common core cryo-EM map at high contour level, centered on the p132 collar (boxed in the inset scheme of the tip). Right: Ribbon representation of a central slice of the collar. The star points to loop 52-60, and the density attributed to LTF_pb1_ is in transparent isosurface representation. (**B**) Left: Isosurface bottom view of the map in (A), slice at the p132 collar level. Right: Bottom view of the four p132 monomers that are not related by the C3 symmetry (colored from light pink to violet and numbered). They interact with two p140 monomers (cyan and blue) and with LTF_pb1_ (transparent densities). The pink/black arrows point to the direction of the LTF_pb1_, and the N and C termini of the proteins are, respectively, indicated by black dots and asterisks. (**C**) Isosurface view at high contour level of Tip-FhuA unmasked and unfiltered cryo-EM map, side view (left) and slice (right). The red arrow points to one of the β-hairpin “leg.” The blue arrow points to the protrusion going through the nanodisc (ND). (**D**) Isosurface view at a lower contour level of Tip-FhuA unmasked cryo-EM map, after a 15-Å low-pass filtering, slice (left) and view from beneath the nanodisc (right). The color code is the same as in Fig. 1. Unattributed densities in (C) and (D) are in white.

The density corresponding to the nanodisc is clearly visible (Fig. 2, C and D), although nanodiscs are heterogeneous in size and in position relative to the tail (fig. S1F). Nanodisc density is not centered with respect to the tail tube axis: Its center of mass is shifted toward the bent fiber, below the density attributed to RBP_pb5,_ under which the structure of FhuA could be fitted (Fig. 2, C and D, and fig. S3D). At low contour level, aligned with the tail tube lumen, a hole in the nanodisc is observed (Fig. 2D), strongly suggesting the presence of a channel at this position. At higher contour level, the tail tube lumen and the nanodisc are filled, and protrusions are visible above and below the nanodisc (Fig. 2C), as if a channel had perforated it. This channel is, however, poorly resolved, probably because of high heterogeneity in that region (fig. S1, F and H).

Limited proteolysis experiments were performed on T5 tails and phages using subtilisin, and resulting particles analyzed by SDS–polyacrylamide gel electrophoresis (SDS-PAGE) and observed by negative stain EM. Within tail assembly, TMP_pb2_ is among the first to be digested (fig. S4, A and D), as it has a tendency to be expelled from the tail by its proximal extremity. This suggests that the protein is contained in the tube in a metastable state. On the contrary, proteins of the tail tip complex are extremely stable. TTP_pb6_ is also very stable with the exception of its decoration immunoglobulin (Ig)–like domain. Upon analysis with negative stain EM, particles appear intact, with the exception of TMP_pb2_ in purified tails (fig. S4, B and E). Infectivity of proteolyzed phages is also only mildly affected, decreasing by only an order of magnitude (fig. S4C). Incubation of tails with FhuA expels TMP_pb2_, making it even more susceptible to the subtilisin action, whereas elements of the tail tip remain resistant to proteolysis despite the vast conformational changes they underwent.

### Core of the tip complex

When comparing the structures of the tip before and after interaction with FhuA, we could observe that they share a common core from the tail tube down to BHP_pb3_ and only start to diverge from BHP_pb3_ distal part. We previously determined a pseudo-atomic resolution structure of T5 tail tube. It is formed of a stack of TTP_pb6_ trimeric rings and exhibits an unusual pseudo sixfold symmetry (hexameric rings being the common tail tube architecture), the TTP_pb6_ gene, resulting in a duplication/fusion of the canonical tail tube domain gene (table S2) (*11*). The tail tube domain is formed by a β-sandwich flanked by an α helix and a long loop (fig. S5A) (*11*). At TTP_pb6_ C terminus, an Ig-like domain decorates the tube, as in many *Siphoviridae* TTPs. Our tail tip structure includes two TTP_pb6_ rings that could be modeled (Fig. 1C). This higher-resolution data confirm our previous modeling of the inter-ring interactions, mostly mediated by the long loops, the N terminus, the linker between the two tail tube domains of TTP_pb6_, and loops of the β-sandwiches (*11*), and have complementary charge surfaces (fig. S6B). The root mean square deviation (RMSD) between TTP_pb6_ structures of the proximal and distal ring is only 0.5 Å over all 464 residues (fig. S5A), suggesting that the interface with the next p140 ring is very similar to that of two TTP_pb6_ rings. The densities of the Ig-like domains are poorly defined (fig. S1C), witnessing a flexibility of this domain with respect to the tube scaffold (*30*).

The tube extends through the tip: After the last TTP_pb6_ ring, it continues with a pseudo-hexameric p140 ring, a hexameric DTP_pb9_ ring, the proximal domains of the BHP_pb3_ trimer forming the last pseudo-hexameric ring of the tube. The structure of these proteins is also based on the canonical tail tube domain (*6*) but differently decorated. Thus, the tube diameter is conserved, although the pitch and the twist between the different rings are different (Fig. 1C). As for TTP_pb6_, the interaction between the rings is mediated mostly by the long loops, the N terminus, and loops of the β-sandwiches (*11*); they also have complementary charged surfaces (fig. S6B). The inner surface of the tube is highly electronegative until DTP_pb9_ (fig. S6A) allows DNA to slide along it (*6*).

p140 pairwise comparison with TTP_pb6_ results in a very high DALI (*31*) *Z* score (fig. S5B), pointing to TTP_pb6_ gene duplication to form p140, despite sequence identity between the two proteins being only 9% (fig. S5C). The main difference between the two proteins is the absence of the Ig-like domain in p140. The p140 ring is surrounded by a larger p132 dodecameric ring, p140 C terminus making direct contacts with a p132 monomer (Figs. 1C and 2, A and B), explaining the need for a decoration-less ring at this position. As suggested by p140 gene position in T5 genome (Fig. 1E), this protein is a bona fide component of its baseplate. p140 and p132 genes are a landmark of the large T5-like phages family only; thus, LTF anchoring would occur differently in other *Siphoviridae*, in particular, in the lambdoid phages. In the *Myoviridae* phage T4, the presence of an additional ring of the TTP-like protein gp54 between the “ring initiator” DTP_gp48_ and the first bona fide TTP_gp19_ rings (*32*) is also observed, and a role in sheath assembly initiation has been proposed (*31*). This additional ring is also not systematically present within the *Myoviridae* family.

We previously determined the crystal structure of DTP_pb9_, showing that the DTP was a common feature to both Gram-positive and Gram-negative infecting siphophages (*22*). In all other phages and tail-like machines, this protein ensures the six-to-threefold symmetry transition between the TTP hexamer and the BHP trimer. Here, however, the DTP_pb9_ ring is sandwiched between two threefold symmetric rings (TTP_pb6_ and BHP_pb3_), which both have a pseudo-sixfold symmetry. This explains the low RMSD between the two DTP_pb9_ monomers that are not related by the imposed threefold symmetry of the map. DTP_pb9_ tail tube domain is decorated with an OB (oligonucleotide-oligosaccharide binding) domain (fig. S5D). The OB domains, as the Ig-like domains, are proposed to interact with carbohydrates at the cell surface and serve to increase infectivity (*4*). Unlike DTPs of siphophages infecting Gram-positive bacteria that serve as a platform to anchor the RBPs (*14*–*16*), DTP_pb9_ does not bind any other protein than those forming the tail tube.

### The collar, p132, and LTF_pb1_

The collar is made of a dodecamer of p132, whose position and structure were previously unknown. The p132 fold belongs to the Ig superfamily (fig. S5E), and a DALI search links it to the N-terminal domain of the baseplate protein upper (BppU; ORF48) of phage TP901-1 (*14*). Ig-like domains in phages are usually decoration domains; here, however, as for BppU, it is a bona fide structural protein that serves to anchor LTF_pb1_. The dodecameric p132 ring completely surrounds the trimeric p140 ring, with p132-p132 and p132-p140 contacts in both p132 and p140 proximal regions and, to a much lesser extent, p132-TTP_pb6_ contacts, mainly mediated through the loops and termini of the three proteins (Figs. 1C and 2, A and B). There are no interactions between p132 and DTP_pb9_ or the Ig-like domain of TTP_pb6_, as determined by PISA (*33*).

Unattributed densities in the lower part of the collar, intertwined between the p132 monomers, most probably belong to LTF_pb1_. However, map quality/connectivity did not allow to unambiguously model it, but it could correspond to its ~50 N-terminal residues. These densities point out of the collar to form the start of the three LTF. The intricate LTF_pb1_-p132 interaction (Fig. 2, A and B) explains that in the fiberless T5-hd1 mutant, which bears a mutation in pb1 gene leading to a truncated protein, the collar is absent, and the p132 protein is not detected by Western blotting. Also, p132 could not be localized within T5 tails using anti-p132 IgGs (*17*). In solution, p132 bears a folded core with very flexible loops and termini (*34*), which could be the target of the IgGs. These loops are mainly unavailable in the phage context, as they are involved in protein-protein interactions (Fig. 2B).

Four consecutive p132 monomers, not related by the threefold symmetry, show high structural similarity (Fig. 2B, and fig. S5, E and F), although their environment is different. There is a symmetry mismatch between LTF_pb1_ 3-fold, p140/TTP_pb6_ pseudo–6-fold, and p132 pseudo–12-fold symmetries. This symmetry mismatch is absorbed by the loops and N termini of p132 monomers. The C terminus and loop 52-60, involved in p132-p132 interactions, are less variable (fig. S5E).

### Closing the tube: BHP_pb3_ and TMP_pb2_

**Fig. 3.**
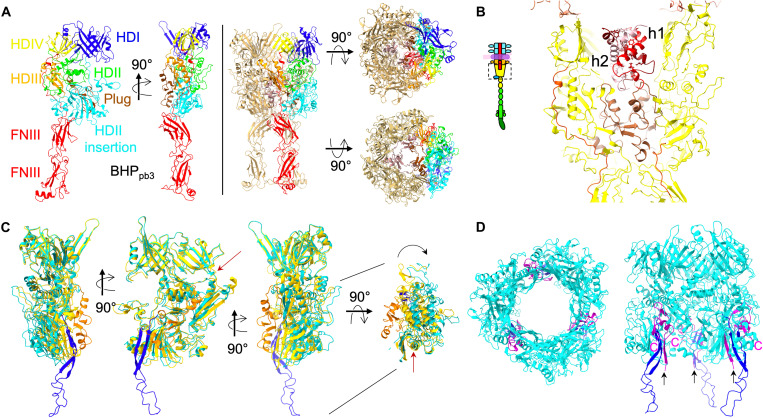
BHP_pb3_ closing and opening of the tube. (**A**) Left: Two side views of a BHP_pb3_ monomer in ribbon representation, with hdI to hdIV domains colored blue, green, orange, and yellow, respectively; the hdII insertion in cyan; the plug in brown; and the C terminus extension, comprising the hdIV-FNIII linker and the two FNIIIs, in red. Right: Side, top, and bottom views of the BHP_pb3_ trimer. One monomer is colored as on the left, and the three plug domains are colored brown. In the bottom view, the FNIIIs have been removed for clarity. (**B**) Central slice through BHP_pb3_ cup, boxed in the inset scheme of the tip (yellow; hdIV-FNIII linker, red; plug, brown) highlighting the 35 resolved residues of a TMP_pb2C_ trimer in different shades of red. Hydrophobic residues of TMP_pb2C_, pointing to the center of the coil are represented in sticks. (**C**) Overlay of BHP_pb3_ before (yellow; plug, orange) and after (cyan; plug, blue) opening of the BHP_pb3_ cone, after superimposition of the whole tip. Three side views 90° apart and a top view are shown. In the top view, hdI and hdIV have been removed to highlight the pivotal movement of the hdII insertion domain. A red arrow points to the long helix of hdIII that acts as a hinge (see also movie S4). The long linker and the FNIIIs have been removed for clarity. (**D**) Top and side views of the open BHP_pb3_ trimer with the same color code as in (C), and TMP_pb2*_ 42 C-terminal residues in magenta. TMP_pb2*_ C termini are indicated (C) as well as the last built residue in N-terminal (T1085, black arrow).

BHP_pb3_ trimer forms the most distal tail tube domain ring of the tube [hub domains (hd) I and IV; Fig. 3A and table S2]. The linker between the two tail tube domains and the long loop of the second tail tube domain have evolved into larger domains (hdII and insertion, and hdIII, respectively; fig. S7, A and B), the first of which is large enough to close the tube via a plug domain (Fig. 3A). A long linker runs along the protein down to the tip of the cone, inserted between two neighboring BHP_pb3_ subunits and contributing to the stability of the closed tube (Fig. 3A). BHP_pb3_ C terminus forms the beginning of the central fiber with two fibronectin domains (FNIII), further stabilizing the closed tube (Fig. 3B) and giving BHP_pb3_ the shape of a trophy cup (Figs. 1, C and D, and 3A). BHP_pb3_ results from a tail tube domain duplication/fusion, as TTP_pb6_. These two duplication/fusion events are clearly independent, however, as the fusion did not occur in the same way in the two proteins (fig. S7B).

Unexpectedly, on unsymmetrized EM reconstructions, an extra density is observed at the base of only one BHP_pb3_ monomer (fig. S3A). Resolution was insufficient to build an atomic model de novo, but secondary structure features could be identified. From the 11 proteins that form the tail and that have been identified by mass spectrometry (MS) (table S3), only p143 has not been located. Its gene position in the tail structural module (Fig. 1E) suggests that it is the tail completion protein, located in the head-to-tail joining region (*4*, *6*). However, we have solved T5 proximal tail region where it could not be attributed to any density. A flexible fit (see the “Protein model building” section) of an AlphaFold2 (*35*) structure prediction of p143 into this extra density was convincing (fig. S3B). Thus, we propose that this unattributed density corresponds to p143. This unusual position for a “Tail Completion Protein” suggests, for p143, a different role than that of completing the tail at its proximal extremity, a role which needs to be elucidated. This density is absent from reconstructions of the tip after interaction with FhuA, suggesting that the protein could detach during the infection process.

The tube lumen is filled with TMP_pb2_. TMP_pb2_ (*20*, *36*), as λ TMP (*37*), undergoes proteolytic cleavage during tail morphogenesis, but it was supposed that the cleaved peptide, TMP_pb2C_, was removed from the final tail assembly, leaving the rest of the protein, TMP_pb2*_, in the tail tube lumen. TMP_pb2_ density is very ill-defined along the tail tube, probably due to a poor interaction network between the predicted coiled-coil (*20*) of TMP_pb2*_ and the wall of the tube, except in the BHP_pb3_ cup (Figs. 1C and 3B). There, we could model TMP_pb2C_ 35 C-terminal residues. Three TMP_pb2C_ copies are coiled in a superhelix, burying hydrophobic residues in its center, interacting closely with BHP_pb3_ plug (Fig. 3B). The TMP_pb2C_ presence in the tail is confirmed by proteomics and liquid chromatography/electrospray ionization/time-of-flight MS (LC-ESI-TOF-MS) (table S3 and fig. S8C) and allowed us to localize TMP_pb2_ cleavage site after R1127 (fig. S5G). This latter is located between the TMP_pb2*_ hydrophobic stretch and a metallopeptidase motif that was shown to have muralytic activity (*20*), separating this enzymatic domain from the rest of the protein. This is reminiscent of the situation in T4, where the cell-puncturing protein gp5 is cleaved during tail assembly between its lysozyme domain and the β helix spike (*38*). It was previously suggested that TMP_pb2_ (*36*), as other phages TMPs [e.g., λ (*39*)], is a hexamer, but our data clearly indicate that only a trimer is present. A trimer of the 20 C-terminal residues of phage 80α TMP was also modeled (*16*), suggesting that this might be a general feature of siphophages. Interactions between TMP_pb2_ and BHP_pb3_ are mainly electrostatic (fig. S6, B and C), unlike in 80α.

### The central fiber and its rearrangement upon receptor binding

BHP_pb3_ C-terminal ~210 residues and pb4 N-terminal ~350 residues were modeled de novo (Fig. 1, C and D). As the resolution of the fiber map drops toward the fiber tip because of its flexibility, the rest of pb4 protein was modeled using flexible fitting of the better resolved equivalent domains built in the Tip-FhuA maps (see below and fig. S1H).

**Fig. 4.**
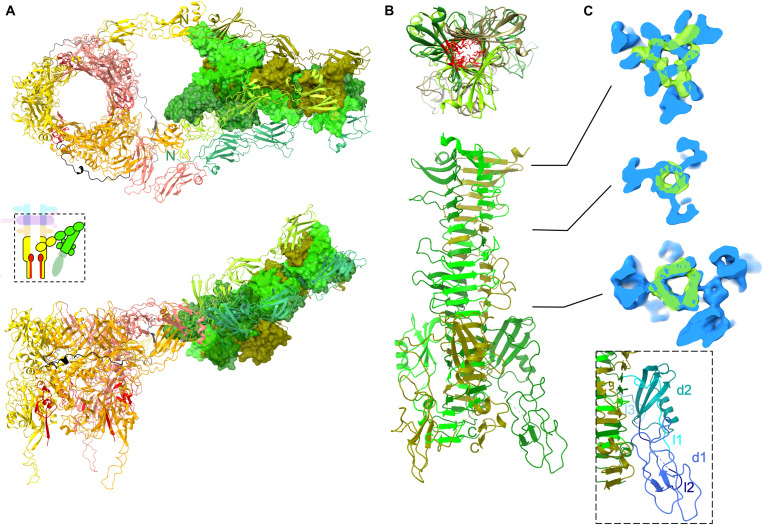
Bending of T5 straight fiber. (**A**) Structure of Tip-FhuA BHP_pb3_, 42 C-terminal TMP_pb2*_ residues, and pb4 (boxed in the inset scheme of the tip). BHP_pb3_ monomers are in gold, orange, and salmon with the hdIV-FNIII linker colored in different shades of gray, pb4 in different shades of green, and TMP_pb2*_ in red. All proteins are in ribbon representation, except for pb4 spike that is in surface representation. Top: Top view. pb4 N termini (N) and BHP_pb3_ C termini (C) are indicated; bottom: side view. (**B**) Top: Top view of pb4 spike. pb4 monomers are in different shades of green. The hydrophobic residues pointing toward the interior of the spike are depicted red and in sticks in one subunit only. Bottom: Side view of pb4 spike. C termini are indicated (C). (**C**) Superimposition of pb4 spike in isosurface view of the tip (green) and Tip-FhuA (blue) maps (unsharpened). Three slices are shown, and their position along the spike is indicated in (B). The map after interaction with the receptor contains the spike decoration domains and the FNIIIs while that before interaction contains only pb4 spike. Inset: pb4 spike decoration domains and linkers are colored in different shades of blue on one pb4 subunit [linker 1 (l1, residues 484 to 547), domain 1 (d1, 548 to 566), linker 2 (l2, 548 to 566), domain 2 (d2, 567 to 618), and linker 3 (l3, 519 to 626)].

The proximal region of the central fiber is made of three strings of five consecutive FNIIIs, two at BHP_pb3_ C terminus and three at pb4 N terminus. It starts as three independent strings; the repulsion between them could be caused by an important negative patch at the surface of the BHP_pb3_ FNIIIs (fig. S6D). BHP_pb3_-pb4 interaction is ensured by two distal loops of the second FNIII of BHP_pb3_ and the N terminus and two proximal loops of the first pb4 FNIII (fig. S8A). After a hinge region, the three pb4 monomers merge to form a 110-Å-long β helix spike, formed of a 24 β strand longitudinal mixed β sheet prism. It has a triangular section with a mean diameter of 20 Å, delineating a very dense and hydrophobic interior (Fig. 4B). β Helix spikes/fibers are very common in phage host recognition or/and perforation apparatus, and a DALI search indeed relates pb4 spike to different phage and tail-like machines spikes/fibers (fig. 8B). Here, however, pb4 does not have a direct role in perforation or host recognition.

The spike is decorated with two small globular domains inserted between β strands 15 and 16 of each pb4 subunit and which are connected by relatively long linkers (Fig. 4B). These domains are not visible in the central fiber map, probably because of the degrees of freedom offered by the linkers. A DALI search links the second small domain to phage spike decoration domains [Protein Data Bank (PDB) codes 7CHU-A, 6TGF-D, 6E1R-A, 5M9F-A, 6NW9-C, 6EU4-A, and 5W6H-A] with a *Z* score of 3.9 to 2.3, RMSD from 2.4 to 3.0 Å over ~45 residues and identity ranging from 4 to 14%. This further illustrates the ability of phages to reuse and exchange functional modules (*40*).

The central fiber ends with RBP_pb5_, but this part of the map is poorly resolved and did not allow building an atomic model of this protein. This 310-Å-long central fiber bears two hinges, one between pb4 last FNIII and the spike and the other at the spike-RBP_pb5_ interface: It introduces some controlled flexibility to this otherwise rigid assembly and may ease RBP_pb5_ exploring space and encountering its bacterial receptor.

Upon RBP_pb5_ binding to FhuA, the central fiber reorganizes: The three FNIIIs strings dissociate, two strings relocate on one side of BHP_pb3_, and the third one on the other side (Fig. 4A and movies S1 and S2). This reorganization of the central fiber is allowed by the long linker that connects BHP_pb3_ hdIV to the first FNIII and which now runs perpendicular to the tail axis along BHP_pb3_. The central fiber bends by ~160° at the level of the hinge between the third pb4 FNIII and pb4 spike: The latter is now surrounded and stabilized by pb4 FNIIIs, which interact with and stabilize pb4 spike small decoration domains (Fig. 4A). pb4 spike also undergoes structural rearrangement, with a different twist of the spike (Fig. 4C). This bending and stabilization of the central fiber bring the tail tube closer to the membrane, orient it correctly, and anchor the tail to the membrane.

### Tube opening and anchoring of the tail to the membrane

As mentioned above, the structures of the tip before and after interaction with FhuA start to diverge from BHP_pb3_ distal domains. More precisely, hdI and hIV overlay remarkably (Fig. 3C and movie S3), with an RMSD of 0.85 Å over 244 residues. However, hdII and hdIII rotate around the long helix of hdIII as a rigid body (the RMSD before and after opening of hdII-III is 0.75 Å over 367 residues), and the plug unwinds in a long β-hairpin (Figs. 3, C and D, and 4A; and movies S3 and S4). These conformational changes result in BHP_pb3_ trimer opening, creating a channel with a constant ~40 Å in diameter from the hdI-hdIV ring to the hdII insertion tip (Fig. 3, C and D, and movie S4). The three β-hairpin legs connect BHP_pb3_ to the nanodisc (Fig. 2, C and D): Their tip is composed of 233-Leu-Phe-Gly-Leu-236, which would insert into the outer leaflet of the lipid bilayer hydrophobic core. Above these hydrophobic residues stand 230-Lys-Lys-Lys-232 and Arg^238^, conferring a strong positive charge to the β-hairpin (fig. S6E). They could interact with the negatively charged phosphate groups of the lipopolysaccharides, further stabilizing the anchoring of the β-hairpin to the membrane.

In the crevice opened at the interface between two BHP_pb3_ subunits, extra densities were identified, in which the 43 C-terminal residues of TMP_pb2*_ could be modeled (Figs. 3D and 4A). These densities merge with the ill-defined densities of BHP_pb3_ β-hairpin, strongly suggesting that TMP_pb2*_ continues toward the nanodisc along BHP_pb3_ β-hairpin, forming with the latter a three-stranded β sheet. TMP_pb2*_ continues with a stretch of nine residues, long enough to reach the nanodisc, followed by a stretch of 46 hydrophobic residues, compatible with two transmembrane helices (fig. S5G), which would insert into the outer membrane and form a channel (fig. S3C). Thus, T5 tail tube is anchored to the outer membrane by both BHP_pb3_ and TMP_pb2*_, in addition to FhuA-RBP_pb5_: It ensures that the tail tube is locked in register with the channel formed by TMP_pb2*_ in the outer membrane.

## DISCUSSION

### Baseplate comparison

As expected, BHP_pb3_ structure partially aligns with other phages and tail-like machine BHPs, with high DALI *Z* scores (fig. S7C): The four hd of the canonical T4 BHP_gp27_ (*38*) are also present in BHP_pb3_, but there is a large insertion in hdII, which closes the tube (Fig. 3A and fig. S7D). In *Myoviridae* and tail-like machines, the tail tube is closed by an OB domain followed by a spike (fig. S7D) (*7*–*9*, *38*). In *Siphoviridae*, there is more diversity for closing the tail tube. The four baseplate structures available to date [phages T5, 80α (*16*), p2 (*15*), and gene transfer agent (GTA) (*41*)] exhibit three different closing modes (fig. S7D): In p2 BHP, two hdII loops pointing toward the lumen of the tube are longer than in *Myoviridae* and close the tube. There, tube opening is induced by an iris-like movement, triggered by Ca^2+^ binding, of hdII-III (*15*). In 80α, the tube is closed by a helix in the C-terminal extension of the BHP that forms a twisted tripod in the trimer lumen (*16*). Last, T5 and GTA tubes are closed by the large hdII insertion (*41*). The two proteins composing GTA BHP, the Hub and the Megatron, align extremely well with BHP_pb3_ (fig. S7, C and E).

Superimposing the *Siphoviridae*-related baseplate structures available, it was notable to observe the remarkable structural superposition of helix 2 of TMP_pb2C_, the resolved helix of TMP_80α_, and helix α1 of the iris/penetration domain of GTA Megatron (fig. S5H). Sequence alignment showed no detectable sequence similarity, and in the case of T5 TMP_pb2C_ and TMP_80α_ C terminus, the interaction with the BHP is via the bottom of the BHP cup (Figs. 1C and 3B). In GTA, helix α1 of the Megatron is proposed to be a pore-lining helix that could insert in the outer membrane to allow the DNA across it, which however cannot be a general feature in *Siphoviridae*.

### T5 trigger for infection and formation of a channel

**Fig. 5.**
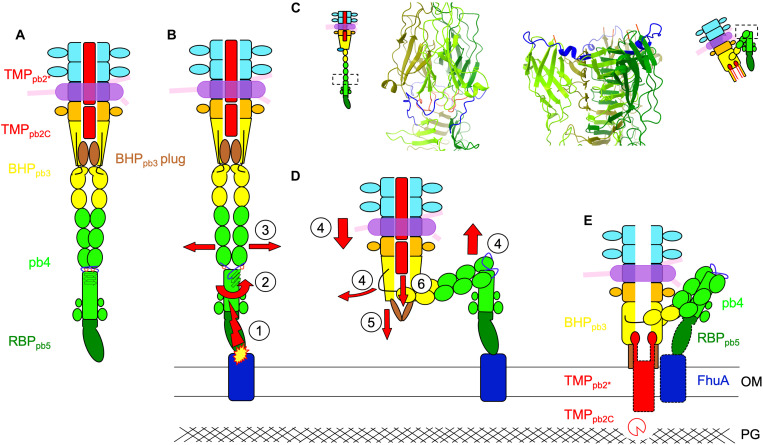
Proposed mechanism of trigger for infection. (**A**) Scheme of T5 tail tip. The hdIV-FNIII linker (black) and the plug (brown) are highlighted in BHP_pb3_, as well as the FNIII-spike linker (blue), loop 224-232 of the third FNIII (salmon), and the orientation of the proximal three β strands of the spike (black arrows) in pb4. (**B**) Following RBP_pb5_-FhuA interaction, a constraint (1) would induce a twisting of the proximal pb4 spike (2), pulling on pb4 FNIII-spike linker. This in turn would destabilize the FNIII string network (3). (**C**) Blow up on pb4 FNIII-spike interface before (left) and after (right) interaction with FhuA. The two spikes are aligned on the middle sheet of the spike (residues 413 to 465). pb4 subunits are in different shades of green, the FNIII-spike linker in blue, and FNIII loop 224-232 in salmon. (**D**) The FNIII string reorganization around pb4 spike induces pb4 bending, brings the tube close to the membrane, and disengages BHP_pb3_ hdIV-FNIII linker (4). This latter event liberates the plug, opening the tube (5) and destabilizing TMP_pb2C_, which is expelled (6). (**E**) BHP_pb3_ plugs refold as a β-hairpin legs and anchor in the outer membrane (OM), TMP_pb2*_ is also expelled, its C termini inserting in the crevice between BHP_pb3_ subunits, its hydrophobic segment inserting in the OM to form a channel. TMP_pb2C_, released in the periplasm, would digest the peptidoglycan (PG). In (E), colored boxes depict proteins that could be modeled (full line) or for which densities are visible (dotted line). TMP_pb2C_, for which no densities is visible but for which we propose a location, is represented as an empty Pacman.

Comparing the structures of T5 tail tip before and after interaction with FhuA, we can propose a mechanism for T5 trigger for infection (Fig. 5). Upon RBP_pb5_ binding to FhuA, a constraint at the RBP_pb5_-pb4 interface occurs (*29*), resulting in the different twist of pb4 spike observed in its proximal part. This twisting of the spike would pull on pb4 FNIII-spike linker, leading to the disruption of the FNIII-spike interaction network (Fig. 5B). The association between this FNIII and the spike is thus loosened; the pb4 FNIII-spike linker reorganizes and stabilizes a new interaction network between the three FNIII strings and the spike (Fig. 5C). This series of events results in the bending of the central fiber, at the level of the FNIII-spike hinge, on the side of the tube, pulling the tube toward the host membrane (Fig. 5D).

To accommodate for the drastic conformational changes of the FNIII strings induced by the bending of the central fiber, BHP_pb3_ hdIV-FNIII linker is pulled and, like a zip, disrupts the interaction network between neighboring BHP_pb3_ monomers and with BHP_pb3_ plug. This then allows the rotation of BHP_pb3_ hdII-III, plug unfolding/refolding, opening of the tube, and anchoring of BHP_pb3_ to the membrane via the β-hairpin legs (Fig. 5D). BHP_pb3_ closed conformation would be stabilized in a metastable state by the assembly process and interaction with its tip partners, TMP_pb2C_ in particular. Open BHP_pb3_ would be of lower energy and would drive the conformational changes underwent by the tip complex upon infection. T5 tip structure was proposed to the CASP14 competition: BHP_pb3_ open structure only was correctly predicted (*42*).

BHP_pb3_ opening disrupts the interaction with TMP_pb2C_, which is expelled from the tube and translocated to the host periplasm where it would locally digest the peptidoglycan; a refolding step could be necessary. TMP_pb2*_ is in turn expelled from the tail tube and anchor its C terminus in the crevice created between BHP_pb3_ monomers upon opening (Fig. 5E). Its hydrophobic segments would then insert in the outer membrane to form a transmembrane channel. These events are thermodynamically favored by hydrophobic segment insertion in the membrane and TMP_pb2*_ alleged metastable state within the tail tube. TMP_pb2_ was shown to be involved in contact points between outer and inner membranes (*43*). Whether TMP_pb2*_ inserts into both the outer and the inner membrane remains to be determined: To form a channel wide enough to allow DNA through would require the six TMP_pb2*_ transmembrane helices. Insertion into the inner membrane could thus occur via another part of the protein and/or the recruitment of host proteins.

The mechanism presented here, by which receptor binding triggers the opening of its tail tube, its anchoring to the host membrane, and formation of a transmembrane channel is, to our knowledge, the first one described for *Siphoviridae*, the most prevalent family of phages. It is furthermore entirely original compared to what was known and described so far for the more complex *Myoviridae* and related tail-like bacterial machines. Our study provides a solid structural basis to further explore the diversity of viral entry mechanisms and their properties. This mechanism was recently complemented by the determination of FhuA-RBP_pb5_ structure, which gives insights into how host recognition (RBP_pb5_ binding to FhuA) triggers infection (*29*).

## MATERIALS AND METHODS

### T5 tail purification

T5 tails were preferred over whole phages for cryo-EM as the former allow thinner ice and no DNA background and, thus, better quality images. Purified tails are able to join filled heads to make infective particles (*44*). *E. coli* strain F cultures at 37°C were infected during the exponential growth phase with the amber mutant phage T5D20*am*30d, which bears an amber mutation in the major capsid protein gene, at a multiplicity of infection of 8. After complete cell lysis [optical density at 600 nm (OD_600_) < 0.15], the cell lysate was incubated with ribonuclease (1 μg/ml), 0.2% chloroform, and 0.5 M NaCl at 37°C for an hour and centrifuged for 20 min at 6000*g* to remove cell debris and unlysed cells. T5 tails were then precipitated from the culture medium by incubation with 8% (w/w) polyethylene glycol (PEG) 6000 overnight at 4°C. The pellet of a 1.5-hour 6000*g* centrifugation was resolubilized in 10 mM tris (pH 7.5), 100 mM NaCl, 1 mM CaCl_2_, and 1 mM MgCl_2_ and purified on a glycerol step gradient (10 to 40%) in the same buffer centrifuged for 2 hours at 20,000 rpm (SW41 rotor). The gradient fractions containing the tails (usually ~10% glycerol), diluted five times in 10 mM tris (pH 7.5), 1 mM CaCl_2_, and 1 mM MgCl_2_, were loaded onto an ion exchange column (HiTrap Q HP 1 ml, GE HealthCare), equilibrated, and washed in the same buffer. The tails were eluted by a 0 to 0.5 M NaCl linear gradient. Purified tails were incubated 30 min with FhuA-loaded nanodiscs at room temperature before preparation of the cryo grids as a longer incubation time leads to a heterogeneous and aggregated sample. The tail/FhuA nanodisc ratio was first screened in negative stain. The chosen ratio included a large majority of tails having interacted with a nanodisc without too high a background in nanodisc.

### FhuA-containing nanodiscs

The gene coding for the MSP1E3D1 (*28*) was cloned in a pET28a plasmid used to transform BL21(DE3) *E. coli*. Protein expression was induced by the addition of 1 mM isopropyl-β-d-thiogalactopyranoside to a 37°C growing Terrific Broth/kanamycin (50 μg ml^−1^) culture when it reached an OD_600_ of 1.2. Cells were harvested 4 hours later, resuspended in a lysis buffer [20 mM NaPO4 (pH 7.4), 1% (w/v) Triton X-100, lysozyme (0.2 mg.ml^−1^), and deoxyribonuclease I (0.2 mg.ml^−1^)] and broken through three to four passages in a microfluidizer (13 kpsi). After clarification of the cell lysate, the supernatant was loaded on a nickel affinity column (HiTrap Chelating HP 5 ml, GE HealthCare) previously equilibrated with 40 mM tris-HCl (pH 8.0), 300 mM NaCl, and 1% (w/v) Triton X-100. The column was then washed with the same buffer, then with 40 mM tris-HCl (pH 8.0), 300 mM NaCl, and 50 mM sodium cholate, then with 40 mM tris-HCl (pH 8.0) and 300 mM NaCl, and then with 40 mM tris-HCl (pH 8.0), 300 mM NaCl, and 10 mM imidazol. The protein was eluted with 40 mM tris-HCl (pH 8.0), 300 mM NaCl, and 300 mM imidazol. The histidine tag was cleaved by incubating the MSP with the Tobacco Etch Virus (TEV) protease overnight in a MSP/TSV 1:10 (w/w) ratio at room temperature in 50 mM tris-HCl (pH 8.0), 0.5 mM EDTA, and 1 mM dithiothreitol and then dialyzed for 2 hours against 20 mM tris-HCl (pH 8.0) and 150 mM NaCl. The cleaved protein was loaded onto the same nickel affinity column equilibrated with 20 mM tris-HCl (pH 8.0) and 150 mM NaCl. Cleaved MSP was recovered in the flow through, concentrated, and loaded onto a size exclusion column (SD75 10/300 GL, GE HealthCare) equilibrated in 20 mM tris-HCl (pH 7.4), 150 mM NaCl, and 0.5 mM EDTA. Fractions of pure protein were pooled, and the protein was concentrated to 2 mg/ml on Amicon concentrator [molecular weight cutoff (MWCO) 100 kDa].

FhuA was produced and purified as described (*23*): *E. coli* AW740 (FhuA31 ΔompF *zcb*::Tn*10* ΔompC) transformed with the pHX405 plasmid, in which the *fhuA*, gene under control of its natural promoter, was grown at 37°C in LB medium supplemented with ampicilin (125 μg ml^−1^), tetracyclin (10 μg ml^−1^), and 2,2′ bipyridyl (100 μM), an iron chelator used to induce FhuA production. After clarification of the cell lysate, total membranes were recovered by ultracentrifugation and solubilized using 50 mM tris (pH 8.0) and 2% (w/w) *N*-octylpolyoxyethylene (Bachem) at 37°C for half an hour. The insoluble material was recovered by ultracentrifugation and solubilized for 1 hour at 37°C using 50 mM tris (pH 8.0), 1 mM EDTA, and 1% (w/w) LDAO (*N*,*N*-dimethyl dodecylamine-*N*-oxide, Anatrace). The solubilized fraction, recovered after ultracentrifugation, was supplemented with 4 mM MgCl_2_ and 5 mM imidazole and loaded on a nickel affinity column (HiTrap Chelating HP 5 ml, GE HealthCare) previously equilibrated with 0.1% LDAO, 20 mM tris (pH 8.0), and 200 mM NaCl and washed with the same buffer. The protein was eluted from the column with 0.1% LDAO, 20 mM tris (pH 8.0), and 200 mM imidazole and loaded onto an ion exchange column (HiTrap Q HP 1 ml, GE HealthCare) equilibrated with 0.05% LDAO and 20 mM tris (pH 8.0). The protein was eluted by a 0 to 1 M NaCl linear gradient.

To produce FhuA-loaded nanodiscs, purified FhuA was incubated with 1,2-dioleoyl-*sn*-glycero-3-phosphocholine solubilized in 100 mM sodium cholate and MSP1E3D1 in a 1:6:360 FhuA:MSP:lipid molar ratio. After 1 hour of incubation at 4°C, detergent was removed by the addition of 0.5 g ml^−1^ BioBeads (Bio-Rad) and incubation on a stirring wheel at room temperature for 2 hours. FhuA-loaded nanodiscs were diluted 6.5 times in 20 mM tris (pH 7.5), 150 mM NaCl, and 5 mM imidazol and further purified on a nickel affinity column equilibrated in the same buffer; eluted with the same buffer containing 200 mM imidazol; desalted on a PD10 (GE HealthCare) desalting column equilibrated in 10 mM tris (pH 7.5), 100 mM NaCl, 1 mM CaCl_2_, and 1 mM MgCl_2_; and concentrated ~40 times on a 50-kDa MWCO Amicon device.

### Cryo-EM sample preparation

Typically, 3.5 μl of T5 tail sample (with or without FhuA nanodisc) was deposited on a freshly glow discharged (25 mA, 30 s) Cu/Rh 300 mesh Quantifoil R 2/1 EM grids and plunge-frozen in nitrogen-cooled liquid ethane using a Thermo Fisher Scientific Mark IV Vitrobot device (100% humidity, 20°C, 5-s blotting time, blot force 0).

### EM data acquisition

Respectively, 3208 and 9608 micrographs (split over two data collections for the latter) were collected for tails alone, and tails were incubated with FhuA nanodisc. Forty-frame movies were acquired on a Thermo Fisher Scientific Titan Krios G3 transmission EM (European Synchrotron Radiation Facility, Grenoble, France) (*45*) operated at 300 kV and equipped with a Gatan Quantum energy filter coupled to a Gatan K2 summit direct electron detector. Automated data collection was performed using Thermo Fisher Scientific EPU software, with a typical defocus range of −1.0 to −3.0 μm and a total dose of 40 e^−^/Å^2^ per movie. A nominal magnification of ×105.000 was used, resulting in a calibrated pixel size at the specimen level of 1.351 Å.

### EM image processing

Frame alignment was performed using Motioncor2 (*46*) keeping, respectively, frames 3 to 30 and 1 to 40 for Tip and Tip-FhuA and applying dose weighting. Contrast transfer function parameters were then determined using Gctf (*47*); manual particle picking was performed with EMAN2/e2helixboxer (*48*). The first picking coordinate was consistently centered on T5 collar and the second one a few hundred angstrom toward BHP_pb3_, along the central fiber (Tip) or the tail axis (Tip-FhuA) (extended data Fig. 2). This “vectorial” picking allowed us to choose and adapt the position of the box along that axis before extraction and proved to be very efficient. All subsequent image processing was performed using Relion (versions 3.0 and 3.1) (*49*). Flowchart of the EM processing pipeline is presented in extended data Fig. 2.

#### Tip

After particle extraction (box size of 340 pixels by 340 pixels) centered 80 Å under the collar and two-dimensional (2D) classification, a homogeneous dataset of 9290 particles was obtained. No 3D classification was performed. Using a 15-Å resolution map determined from a previous cryo-EM data collection (*50*) as an initial model, a C3 reconstruction of the tip was calculated, from the second TTP_pb6_ ring to the beginning of the central fiber. After masking and sharpening, the overall estimated resolution of the map reached 3.53 Å [Fourier Shell Correlation (FSC)_0.143_]. A new set of particles (box size of 400 pixels by 400 pixels) was extracted after a 150-pixel coordinate shift on the *z* axis, toward RBP_pb5_. A 15-Å low-pass–filtered initial model was generated from the newly extracted particles using relion_reconstruct tool and determined a C3 reconstruction of the central fiber from BHP_pb3_ to the beginning of RBP_pb5_. After masking and sharpening, the overall estimated resolution of the map reached 4.2 Å (FSC_0.143_). An additional map of the full tip, of overall lower resolution (FSC_0.143_ 3.88 Å), was also calculated to be able to fit the entire tip model but was not used for model building. Further image processing was necessary to obtain a map including the monomeric p143 protein; refined particles from the tip reconstruction were reextracted (box size of 200 pixels by 200 pixels) and recentered on the lower part of BHP_pb3_, on the side of which p143 is located. After reclassification/selection, symmetry relaxation, and a new run of 3D refinement using suitable masking, a nonsymmetrized map of the central part of the tip was obtained, where densities for the monomeric p143 are visible.

#### Tip-FhuA

After particle extraction (box size of 340 pixels by 340 pixels) and extensive 2D and 3D classifications, a homogeneous dataset of 20,349 particles was obtained. As an initial model, an 8-Å resolution map (low-pass–filtered at 15 Å) obtained from a previous cryo-EM data collection was used, and a reconstruction of the full nonsymmetrized tip after interaction with FhuA nanodisc (full tip FhuA) was calculated. After masking and sharpening, the overall estimated resolution of the map reached 4.3 Å (FSC_0.143_). Signal subtraction was then performed to enhance the resolution of specific parts of the structure. On the basis of the previously determined reconstruction, two soft masks were created, for the bent fiber only and for the C3 open tube. After reextraction and 3D reconstruction, the overall quality of these two areas greatly improved, with overall estimated resolution of 4.3 and 3.60 Å, respectively, for the bent fiber and the open tube.

#### Tip/Tip-FhuA common core

To improve the resolution of the tip common core (TTP_pb6_, p132, p140, and DTP_pb9_), and because we observed that it was invariant whether the tails were incubated with FhuA nanodisc, particles from all three datasets were merged. A soft mask was created using a 20-Å resolution model generated with Chimera tool molmap, using a previously built atomic model (see the “Protein model building” section below) containing two TTP_pb6_ trimers, a p140 trimer, a DTP_pb9_ hexamer, and a p132 dodecamer and used to perform signal subtraction on the merged particles. Subtracted particles were then refined to obtain a better C3 reconstruction for the tip common core, whose resolution reached an overall estimated value of 3.4 Å, allowing us to build slightly more accurate atomic models for TTP_pb6_, p140, DTP_pb9_, and p132 proteins. Efforts to specifically isolate and align the ill-defined RBP_pb5_/FhuA-RBP_pb5_ parts of the maps did not result in any improvement, probably due to the small size of the protein and/or the low number of particles. For every reconstruction, a local resolution map was calculated using Relion built-in local resolution tool (extended data Fig. 1, C and H).

### Protein model building

Atomic protein models were built into the different cryo-EM maps (table S4) using the Coot software (*51*) by tracing the protein sequence into the densities and were then iteratively refined alternating Coot manual refinement and PHENIX (*52*) real space refine tool until convergence. p140, p132, BHP_pb3_, and TMP_pb2C_ models were built ab initio. For TTP_pb6_ and DTP_pb9_ models, existing x-ray models [5NGJ (*11*)/4JMQ (*22*)] were used as a starting point and were refined into the EM maps. MolProbity (*53*) was used for model quality assessment. The densities corresponding to the BHP_pb3_ β-hairpin legs (residues ~225 to 245) in the Tip-FhuA map are poorly resolved with regard to the rest of the protein. As a consequence, we only propose a likely model for BHP_pb3_ β-hairpins, which should be considered with caution.

For p143, we used an AlphaFold2 (*35*)–predicted model as a starting point, which was fitted into the corresponding densities. Notably, AlphaFold2’s level of confidence was not optimal throughout the whole sequence, which could explain the partial fit of the initial model. It was though coherent regarding the global shape and size. We then used a combination of Flex-EM (*54*)/Namdinator (*55*) (flexible fitting) and PHENIX (*52*) (real space refine), in an iterative way, to obtain a better model for this protein, with a convincing fit of most of its secondary structures.

### Proteomics based on high-performance LC/ESI orbitrap

T5 tail proteins were stacked on the top of a 4 to 12% NuPAGE gel (Invitrogen) and stained with R-250 Coomassie blue. Gel bands were subjected to digestion using modified trypsin (Promega, sequencing grade) as previously described (*56*). Peptides were analyzed by online nano–LC tandem MS (LC-MS/MS) (UltiMate 3000 RSLCnano and QExactive Plus, Thermo Fisher Scientific) with two replicates per sample. Peptides were sampled on a 300 μm by 5 mm PepMap C18 precolumn and separated on a 75 μm by 250 mm C18 column (PepMap, Dionex). The nanoLC method consisted of a 120-min gradient at a flow rate of 300 nl/min, ranging from 5 to 37% acetronitrile in 0.1% formic acid for 114 min, before reaching 72% acetronitrile in 0.1% formic acid for the last 6 min.

Spray voltage was set at 1.6 kV; heated capillary was adjusted to 270°C. Survey full-scan MS spectra [mass/charge ratio (*m*/*z*) = 400 to 1600] were acquired with a resolution of 70,000 after accumulation of 10^6^ ions (maximum fill time, 200 ms). The 10 most intense ions were fragmented by higher-energy collisional dissociation after accumulation of 10^5^ ions (maximum fill time, 50 ms). LC-MS/MS data files were processed using MaxQuant, version 1.5.1.2 (*57*). Spectra were searched against the UniProt database and frequently observed contaminants database. The minimum number of unique peptides is 1. Matching between runs option was activated. Proteins identified in the reverse and contaminant databases, or with less than two razor + unique peptides, or exhibiting less than six intensity based absolute quantification (iBAQ) values in one condition were discarded. After log_2_ transformation, iBAQ values for the remaining proteins were normalized by condition-wise centring, missing values were imputed for each injected sample as the 2.5 percentile value, and statistical testing was conducted using Welch’s *t* test.

### Liquid chromatography/electrospray ionization mass spectrometry

To measure the accurate mass of the tail proteins, the T5 tails were diluted 2:3 in 0.1% trifluoroacetic acid (TFA) to obtain a final tail concentration of 0.20 to 0.22 μM and were analyzed using ESI-TOF mass spectrometer (6210 instrument, Agilent Technologies) coupled to an LC system (1100 series, Agilent Technologies). The instrument was calibrated using tuning mix (ESI-L, Agilent Technologies). The following instrumental settings were used: gas (nitrogen) temperature, 300°C; drying gas (nitrogen), 7 liters min^−1^; nebulizer gas (nitrogen), 10 psig; Capillary voltage, 4 kV; fragmentor, 250 V; skimmer, 60 V; and peak-to-peak voltage (octopole radio frequency), 250 V. The high-performance LC (HPLC) mobile phases were prepared with HPLC-grade solvents. Mobile phase A composition was 5% acetonitrile (ACN) and 0.03% TFA. Mobile phase B composition was 95% ACN and 0.03% TFA.

Eight microliters of each sample (1.6 to 1.8 pmol) was first desalted on-line for 3 min with 100% of mobile phase A (flow rate of 50 μl/min) using a C8 reverse phase micro-column (Zorbax 300SB-C8, 5 μm, 5 mm by 0.3 mm, Agilent Technologies). The sample was then eluted with 70% of mobile phase B (flow rate of 50 μl/min), and MS spectra were acquired in the positive ion mode in the 300 to 3000 *m*/*z* range. Data were processed with MassHunter software (v. B.02.00, Agilent Technologies) and GPMAW software (v. 7.00b2, Lighthouse Data, Denmark).

## Supplementary Materials

### This PDF file includes:

Figs. S1 to S8 Tables S1 to S4 Legends for movies S1 to S4 References
