# Supplementary material for: Structural basis of bacteriophage T5 infection trigger and E. coli cell wall perforation
Source: Sci Adv. 2023 Mar 24;9(12):eade9674. doi: 10.1126/sciadv.ade9674 (PMC10038345; doi:10.1126/sciadv.ade9674)
Supplement: Supplementary file 1 — Figs. S1 to S8 Tables S1 to S4 Legends for movies S1 to S4 References [file sciadv.ade9674_sm.pdf]

Science Advances
AAAS

Supplementary Materials for

Structural basis of bacteriophage T5 infection trigger and *E. coli* cell wall perforation

Romain Linares *et al.*

Corresponding author: Cécile Breyton, cecile.breyton@ibs.fr

Sci. Adv. **9**, eade9674 (2023)
DOI: 10.1126/sciadv.ade9674

The PDF file includes:

Figs. S1 to S8
Tables S1 to S4
Legends for movies S1 to S4
References

Other Supplementary Material for this manuscript includes the following:

Movies S1 to S4

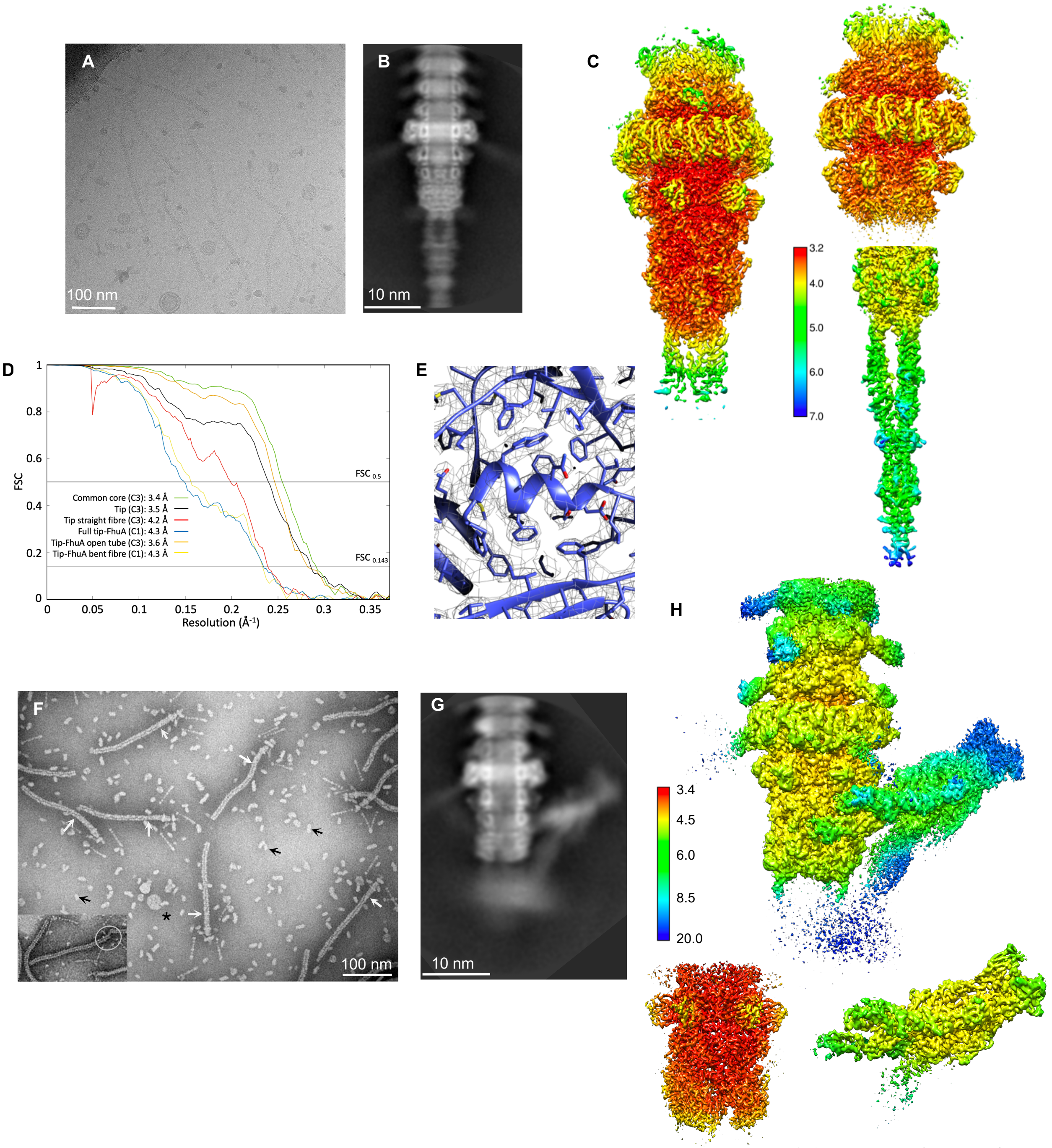

Figure S1: Structure of T5 tail tip before and after interaction with FhuA in nanodisc. (**A**) Cryo-EM image of T5 tails. (**B**) A 2D class average of T5 tail tip. (**C**) Local resolution maps, as determined by Relion of the tip (left), the Tip/Tip-FhuA common core (top right, from TTP_{pb6} to the BHP_{pb3} top part, map obtained gathering particles from both Tip and Tip-FhuA datasets) and of the tip straight central fibre (bottom right). All maps were calculated with a C3 symmetry and the key (in Å) is the same for the three maps. Resolution is the highest at the centre (3.2 Å) and falls off radially to ~7 Å at the tip of TTP_{pb6} Ig-like domain. Because of the flexibility of the central fibre and of the tube, resolution also drops rapidly along the tube, above the first TTP_{pb6} ring and downwards along the central fibre. (**D**) Fourier shell correlation plot for the six maps presented in C and H. $FSC_{0.5}$ and $FSC_{0.143}$ cutoffs are indicated, as well as the estimated resolution ($FSC_{0.143}$) for each map. (**E**) Close up view of an α-helix of p140 monomer model, built using Tip/Tip-FhuA common core map. (**F**) Large field, negative stain EM image of T5 tails incubated with FhuA-nanodiscs. The background is filled with FhuA-nanodiscs, mainly seen lying on the side (black arrows) but also from the top (*). T5 tails are partially emptied from TMP_{pb2}. White arrows point to the empty/filled limit in the tails. Inset: T5 tails incubated with detergent-solubilised FhuA. In those conditions, TMP_{pb2} has been completely ejected and the central fibre is not visible. (**G**) A 2D class average of Tip-FhuA. (**H**) Local resolution maps, as determined by Relion, of the C1 full Tip-FhuA (top), C1 bent fibre (BHP_{pb3} FNIII, pb4, bottom right) and C3 open tube (DTP_{pb9}, BHP_{pb3} ~700 N-terminal residues, TMP_{pb2^*} 43 C-terminal residues, bottom left). The key (in Å) is the same for the three maps.

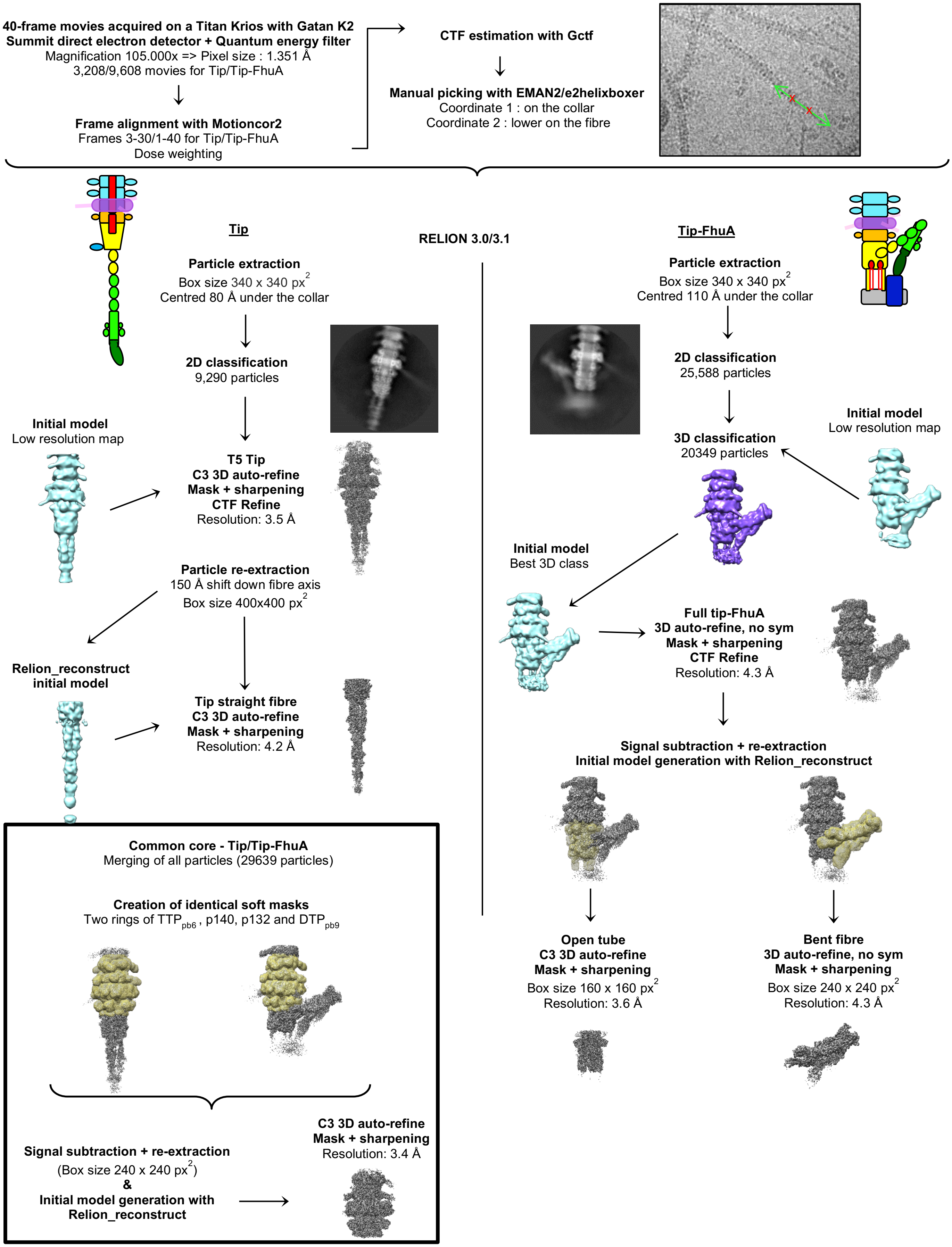

Figure S2: Flowchart of the EM processing pipeline for T5 Tip and Tip-FhuA. Common steps for the Tip/Tip-FhuA datasets are framed in blue. Initial models are in blue, 3D classes in purple, soft masks in yellow and 3D refine in grey. Top right: vectorial picking of the tip particles on a cryo-EM image of a T5 tail tip.

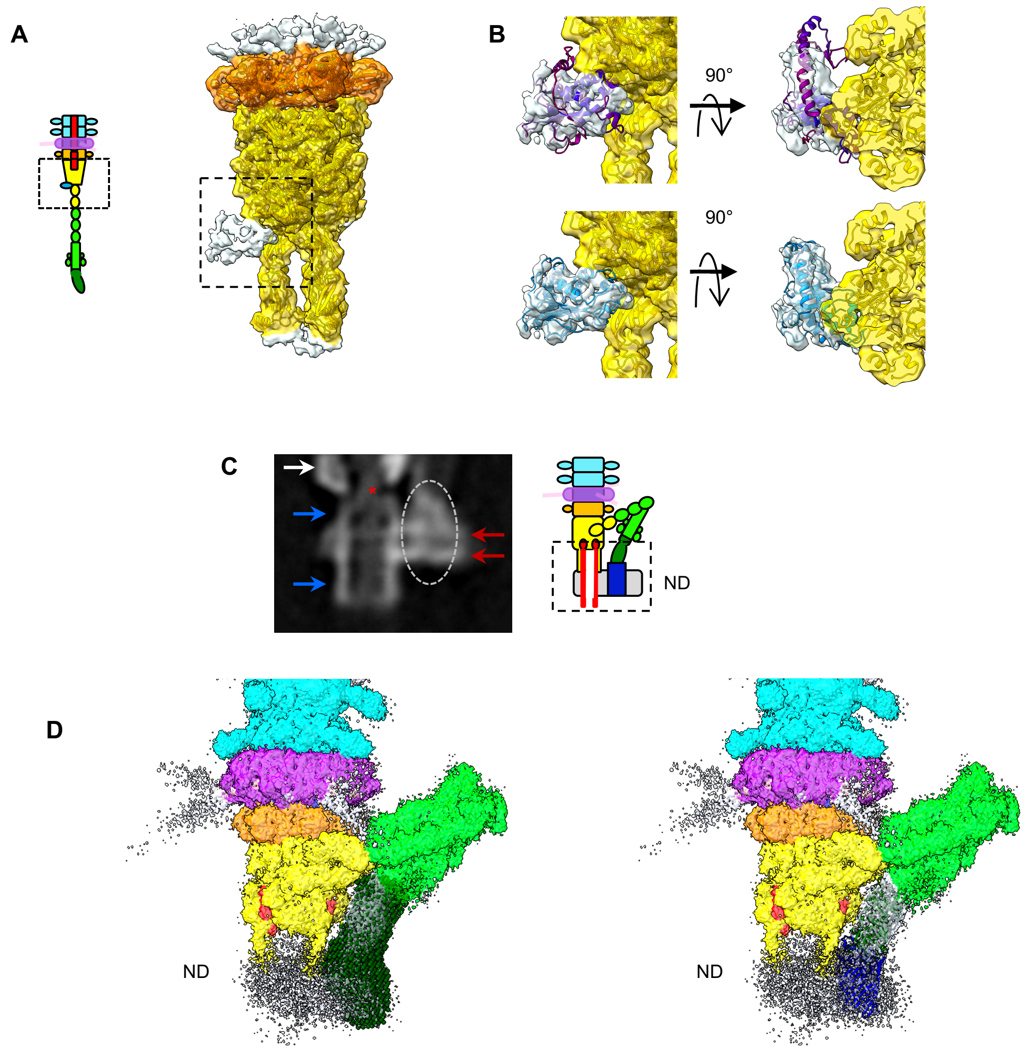

Figure S3: Fit of p143, FhuA and RBP$_{pb5}$ in unattributed densities. (A) Isosurface view of an unsymmetrised cryo-EM map of the tip (50% transparency), with models for the BHP$_{pb3}$ trimer (yellow) and the DTP$_{pb9}$ hexamer (orange) fitted in it. Additional densities at the base of BHP$_{pb3}$, corresponding to a monomeric protein, are clearly visible (dotted box). (**B)** Enlargement of the dotted box in A, with a fit of the Alphafold2 predicted structure for p143 before (top, coloured according to the pLDDT (high: blue, low: magenta)) and after (bottom, light blue model) flexible fitting. Left: side view, right: top view from a slice. (**C)** 2D Slice of a low resolution EM reconstruction from Tip-FhuA showing the bottom of BHP$_{pb3}$ (white arrow), the nanodisc bilayer (red arrows) and what appears to be a cylindrical structure connected to BHP$_{pb3}$ and spanning the nanodisc (blue arrows), probably the channel or part of it. Densities corresponding to TMP$_{pb2*}$ can also been seen inside the tube/channel (red asterisk). The position of FhuA is marked by a white dotted oval. (**D**) Left: Fit of a SANS envelop of the FhuA-pb5 complex (*24*)(dark green beads) into the densities prolonging pb4 spike and merging into the nanodisc. Right: Fit of FhuA-RBP$_{pb5}$ (PDB 8B14) in the same map (*29*)(FhuA: blue and RBP$_{pb5}$ green ribbon). The map is that of Tip-FhuA C1 filtered and masked reconstruction, coloured as in figure 1 and 50% transparent. Unattributed densities are white. ND indicates the position of the nanodisc.

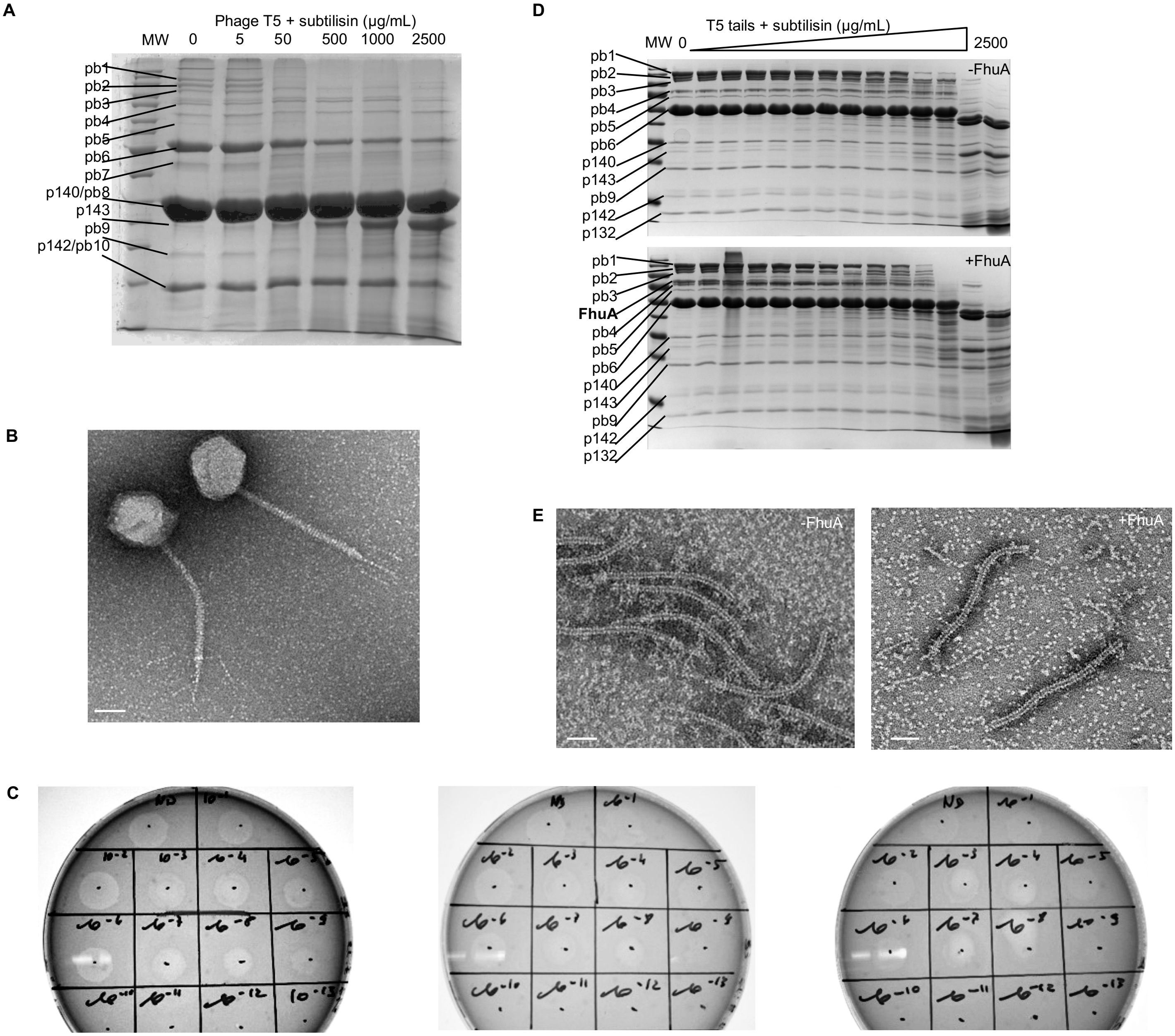

Figure S4: Limited proteolysis of phage T5 (A, B, C) and T5 tails (D, E). (A) Phage T5 was incubated with subtilisin (0, 5, 50, 500, 1000, 2500 µg/mL) for 1h at room temperature. The reaction was stopped by the addition of 10 mM PMSF, and phage ghosts (*17*) were prepared for migration on 12% SDS-PAGE. Molecular weight markers: 200, 150, 120, 100, 85, 70, 60, 50, 40, 30, 25, 20, 15, 10 kDa. (**B)** Negative stain EM image of phage T5 after 1h incubation with 2.5 mg/mL subtilisin. (**C)** Titration of unproteolysed T5 (left), T5 incubated 1h with 50 µg/mL (middle) and 2.5 mg/mL (right) subtilisin on a lawn of *E. coli* strain F in soft Agar. (**D)** Upper panel: Purified T5 tails were incubated with subtilisin (0, 0.1, 0.3, 0.6, 1, 2, 3, 5, 7.5, 10, 20, 30, 1000, 2500 µg/mL). Lower panel: T5 tails were pre-incubated with LDAO-solubilised FhuA for 1h at room temperature (final LDAO concentration 0.05%), before being incubated with subtilisin. Molecular weight markers: 180, 130, 100, 70, 55, 40, 35, 25, 15, 10 kDa. (**E)** Negative stain EM images of T5 tails (right) and T5 tails incubated with FhuA (left) incubated with 2.5 mg/mL subtilisin. Subtilisin is clearly visible in the background. Bar: 50 nm.

A

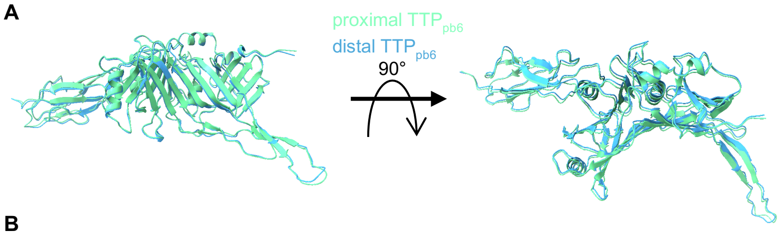

B

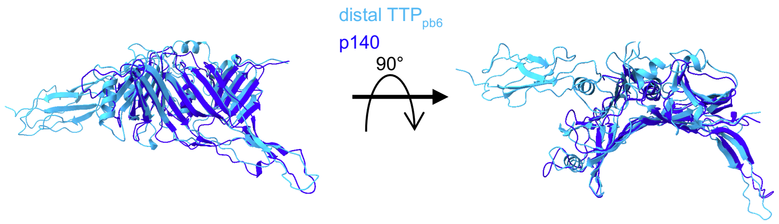

D

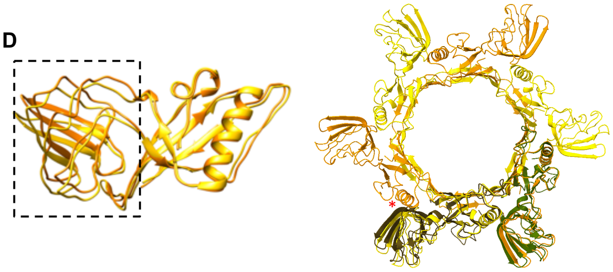

E

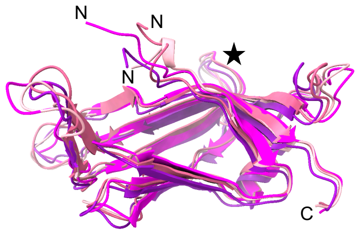

C

```
pb6   MSLQLLRNTRIFVSTVKtghnktNTQEI-LVQDdISWGQDSNSTDITVNEAGprPTRGSK
ident |   | |   |                                            |
p140  MFYSLMRESKIVIEYDG------RGYHFdALSN-YDASTSFQEFKTLRRTIH-nRTNYAD

pb6   RFNDSLNAAEWSFSTYILPYkdkntskqiVPDYMLWHALSSgrainlegttGAHNnatnF
ident            |
p140  SIINAQDPSSISLAINFSTT---------LIESNFFDWMGF----------TREG----N

pb6   MVNFK-DNSYHELAMLHIYILTD-KTWSYIDSCQINQAEVNVDIeDIGRVTWSGNGNQLI
ident            |  |   ||        |   |         |   |
p140  SLFLPrNTPNIEPIMFNMYIINHnNSCIYFENCYVSTVDFSLDK-SIPILNVGIESGKFS

pb6   PLdEQPFdpdqIGIDdetymtIQGSYIKnKLTILKIKdmdtnkSYDIPITGGTFTINNNI
ident             |                              |
p140  EV-STFR----DGYT----itQGEVLPY-SAPAVYTN-----sSPLPALISASMSFQQQC

pb6   TYLTPN---VMSRVTIPIGSFTGAFELTGSLTAYLNDKSlgsmELYKDLiktlkvvNRFE
ident                               |  |          |  |         |
p140  SWREDRnifDINKIYTNKRAYVNEMNASATLAFYYVKRLvgdkFLNLDP------eTRTP

pb6   IALVLGgeydderpAAILVAKQAHVNiPTIETDDVLGTSVEFKAIPSdldagDEGYLGFS
ident                       |          |                  |     |
p140  LIIKNK--------YVSITFPLARIS-KRLNFSDLYQVEYDVIPTAD----sDPVEINFF

pb6   SKYTrttinnlivngdgatdavtaitvksagnvttlnrsatlqmsvevtpssarnkevtw
ident
p140  GERK--------------------------------------------------------

pb6   aitagdaatinatgllradasktgavtveatakdgsgvkgtkvitvtagg  464
ident
p140  --------------------------------------------------  298
```

F

DALI Z-score ↓ / rmsd (Å) →	p132-1	p132-2	p132-3	p132-4
**p132-1**	140	1.5	1.7	1.5
**p132-2**	20.2	140	1.5	1.4
**p132-3**	20.4	19.9	140	1.5
**p132-4**	19.6	20.3	20.6	139

G

```
       970        980        990       1000
GSLDTTSMIA SGMQTVASMI QYSTSQQVSA IDQAIAAEQK

      1010       1020       1030       1040
RDGKSEASKA KLKKLEAEKL KIQQDAAKKQ IIIQTAVAVM

      1050       1060       1070       1080
QAATAVPYPF SIPLMVAAGL AGALALAQAS SASGMSSIAD

      1090       1100       1110       1120
SGADTTQYLT LGERQKNVDV SMQASSGELS YLRGDKGIGN

      1130       1140       1150       1160
ANSFVPRAEG GMMYPGVSYQ MGEHGTEVVT PMVPMKATPN
              *  *  *   *  ** *  ** *     *
      1170       1180       1190       1200
DQLSDGSKTT SGRPIILNIS TMDAASFRDF ASNNSTAFRD
      1210      1219
AVELALNENG TTLKSLGNS
```

H

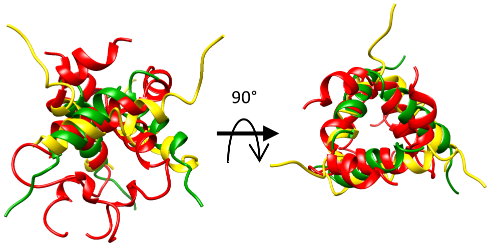


```
T5-TMPpb2      NSTAFRDAVELALNE-
80α-TMP        DALTSIVNGRNAKRNS
GTA-Megatron   AAGAAIGGGFGGTVL-
```

Figure S5: Structural analysis of p140, DTPpb9, p132 and TMPpb2C. Comparison between upper and lower TTPpb6 (**A**) and between lower TTPpb6 and p140 (DALI Z-score of 22.6, with an rmsd over 282 residues of 2.6 Å) (**B**). Side (left) and top (right) views. (**C**) Sequence alignment between TTPpb6 and p140. Residues from aligned structures are uppercase. Identical residues are highlighted with a vertical bar. (**D**) Left: Superimposition of the two DTPpb9 subunits unrelated by symmetry, aligned on the tail tube domain (rmsd 1.1 Å over all 204 residues, 0.8 Å over the 112 residues of the tail tube domain), the OB domain is framed by a rectangle. Right: DTPpb9 hexameric ring, in which two subunits of the asymmetric unit are superimposed, on the tail tube domain, with the crystal structure of DTPpb9 (in green). The OB domain of monomer 2 is making contact with the tail tube domain of monomer 1 (*). (**E**) DALI superposition of the four p132 of the asymmetric unit. The N- and C-termini are indicated and the star marks loop 52-60. (**F**) Table

summarising the pairwise superpositions of the four p132 subunits of the asymmetric unit. In the diagonal in grey is the number of residues built for each subunit, above the diagonal the rmsd in Å and below the DALI Z-score. DALI search links the p132 fold to the N-terminal domain of the Baseplate Protein Upper (BppU, ORF48) of phage TP901-1 with a Z-score of 5.9 and a rmsd of 4.0 Å over 99 residues and 9% identity. (**G)** Sequence analysis of the TMP_{pb2} 259 C-terminal residues. Bold: predicted coiled-coil region from COILS; underlined: predicted transmembrane domain from PSI-PRED and TMHMM; green: peptides identified in proteomics; blue: Zinc carboxypeptidase motif, with conserved residues from Prosite analysis indicated with a star; purple arrow: cleavage site, separating TMP_{pb2*} and TMP_{pb2C}. The red box corresponds to TMP_{pb2C} 35 modelled residues from the Tip map, those pointing towards the centre of the coil are underlined. The magenta box correspond to TMP_{pb2*} C-terminus 42 modelled residues in Tip-FhuA map. (**H)** Overlay of T5 TMP_{pb2C} (red), TMP_{80a} (green) and the N-terminal helix of GTA Megatron (yellow) when superimposing the BHP trimers of each baseplate. Sequence alignment of the aligned helices, with the hydrophobic residues pointing towards the centre of the coil underlined.

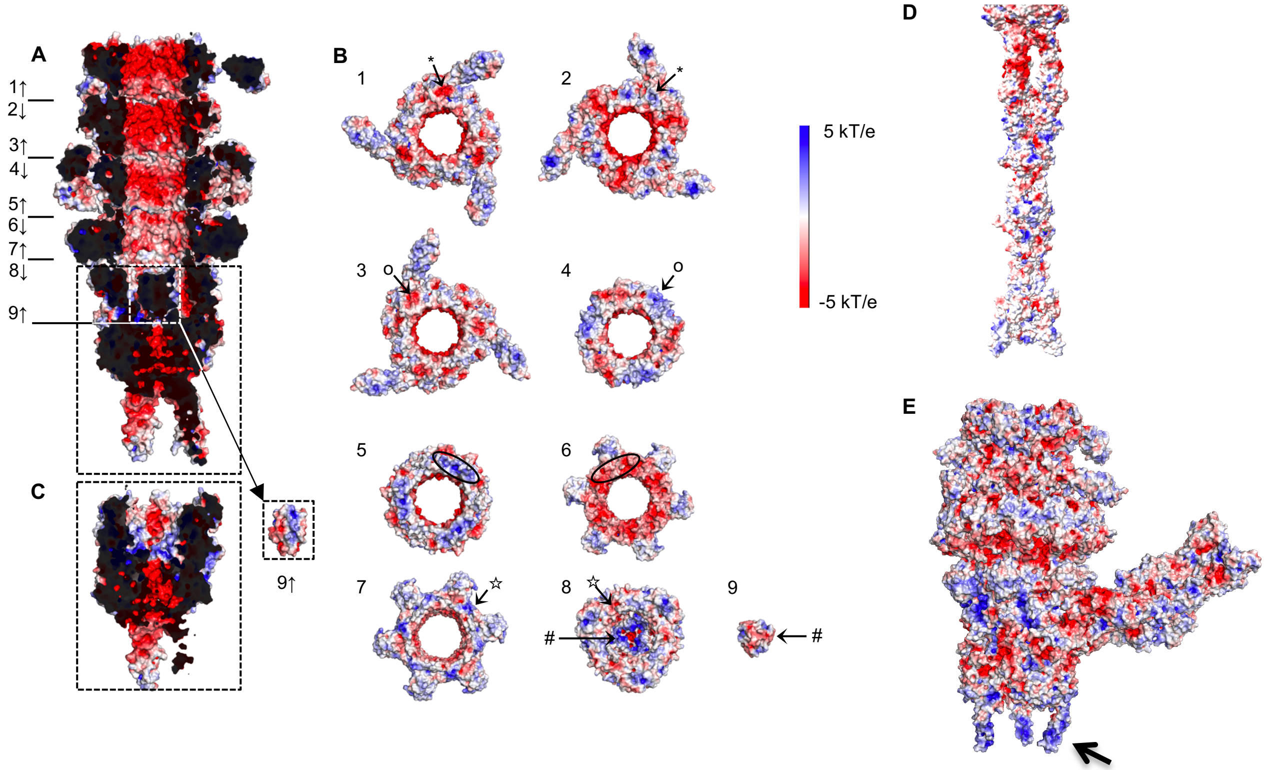

Figure S6: Electrostatic charge distribution. (**A)** Interior of T5 tail tube, (**B)** complementary interfaces of the different rings, **(C)** closed BHP_{pb3} in which TMP_{pb2c} has been removed *in silico* and is shown on the right side, (**D**) tail tip before and after interaction with FhuA**.** The arrow points to one of the highly charged three β-hairpins 'legs'. The position of the views in B is indicated by a number in A, and complementary charge patches are noted by symbols. Electrostatic charge distribution was calculated from the APBS plugin of either PyMol or ChimeraX.

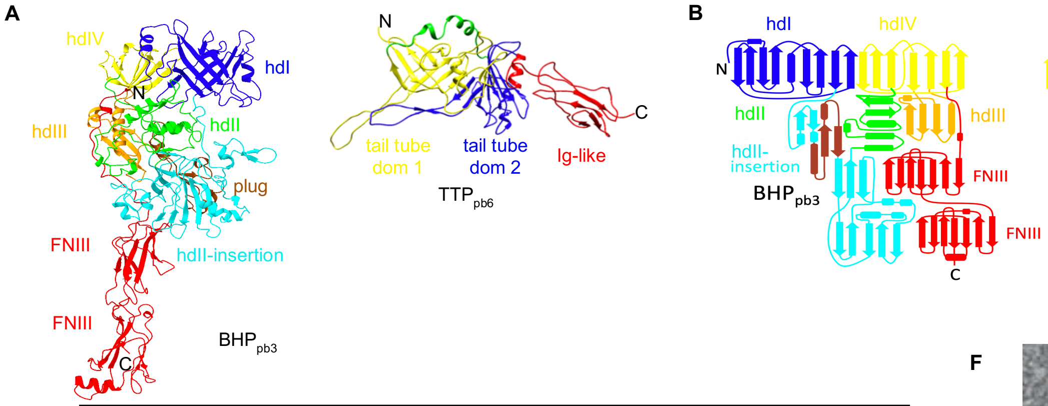

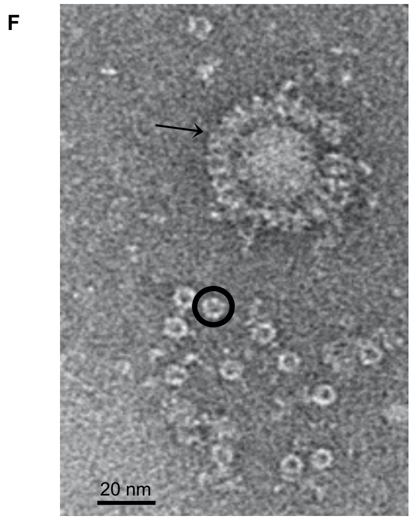

C

PDB code	Dali Z-score	rmsd (Å)	Aligned/total residues	% id	Protein
6TEH	13.9	5.5	354/439	12	* GTA Megatron gp15
6J0M	13.6	3.7	292/535	13	PCV VGRG pcv8
1K28	13.5	3.5	306/364	10	T4 BHP gp27
6U5H	12.9	3.9	295/318	10	Pyocin BHP
6V8I	11.1	3.9	288/387	12	* 80α BHP gp59
2P5Z	10.8	3.7	257/361	11	T6SS VGRG
6TEH	9.6	3.3	178/292	16	* GTA Hub gp13
6RBK	8.8	4.0	286/504	11	AFP VGRG afp8
2WZP	6.2	4.4	224/375	10	* p2 BHP ORF15

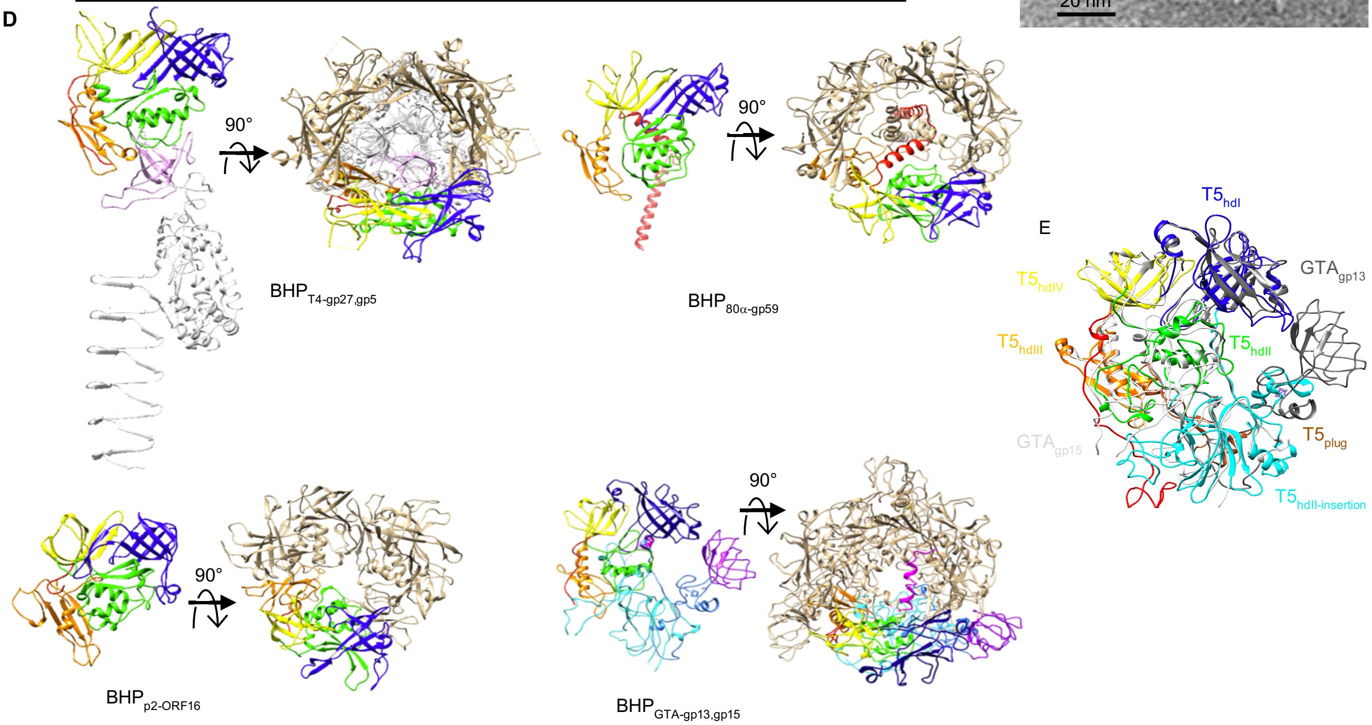

Figure S7: Structural analysis of BHP$_{pb3}$. **(A)** Ribbon representation in the same side view orientation of TTP$_{pb6}$ (left) and BHP$_{pb3}$ (right) and **(B)** topology diagram of the two proteins. BHP$_{pb3}$ hdI-VI are coloured blue, green, orange and yellow, respectively, the hdII insertion in cyan and the two FNIII in red. The same colour code was used for structurally homologous domain in TTP$_{pb6}$. The link between the two tail tube domains in BHP$_{pb3}$ and TTP$_{pb6}$ is topologically different in the two proteins: it connects the C-terminus of the first domain to the N-terminus of the

second one in TTP_{pb6}, and the N-terminus the first domain to the C-terminus of the second one in BHP_{pb3}. N- and C-terminus are indicated. (**C)** DALI search using BHP_{pb3} without its FNIIIs (716 residues) as a bait. Asterix point to *Siphoviridae* BHPs. (**D)** Side views of monomeric BHP proteins of T4-gp27, 80α-gp59, p2-ORF16 and GTA-gp13-15 with the same colour code as in A, and top views of the trimeric complex, with one monomer coloured. In T4, gp5 was added, with the OB domain that closes the tube coloured in pink and the needle in white. Note that GTA BHP is composed of two proteins, the Hub (gp13) and the Megatron (gp15). The Hub protein comprises hdI (darker blue), part of the insertion domain (darker cyan) in which is inserted an OB domain (purple), while the Megatron comprises hdII (green), part of the insertion domain (cyan), hdIII (orange), hdIV (yellow) and an N-terminal "iris helix" (magenta). (**E)** Side view superimposition of BHP_{pb3} (coloured) with GTA-Megatron (light grey) and GTA-Hub (dark grey) in ribbon representation. **F.** Negative stain EM images of purified BHP_{pb3} showing isolated monomers coexisting with either free trimers seen in top views (circled), or that aggregate around impurities, seen in side views (arrow). The dimensions of the trimer (hight: 45 nm, diameter: 9 nm) correspond to those of the BHP_{pb3} trophy cup. In the top views, six subdomains can be distinguished (see circled particle).

A

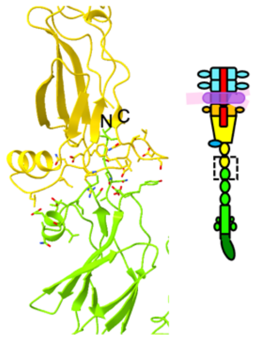

B

PDB code	Dali Z-score	rmsd (Å)	Aligned/total residues	% id	Protein
**6U9E**	3.9	3.0	72/133	17	PDPA-VGRG complex<br>Part of *Francisella* T6SS puncturing spike
**6SJL**	3.3	5.4	100/324	14	VGRG<br>*E. coli* T6SS puncturing spike
**6J0M**	3.3	4.7	74/535	15	PVC8<br>PVC puncturing spike
**5NXH**	2.9	7.0	139/546	6	T4 long tail fibre gp34$^{744-1289}$
**6EU4**	2.8	10.0	111/587	5	VB_ABAP_AS12 gp42<br>*Acinetobacter* phage tail RBP spike
**6C15**	2.7	8.5	93/374	1	Pyocine tail fibre

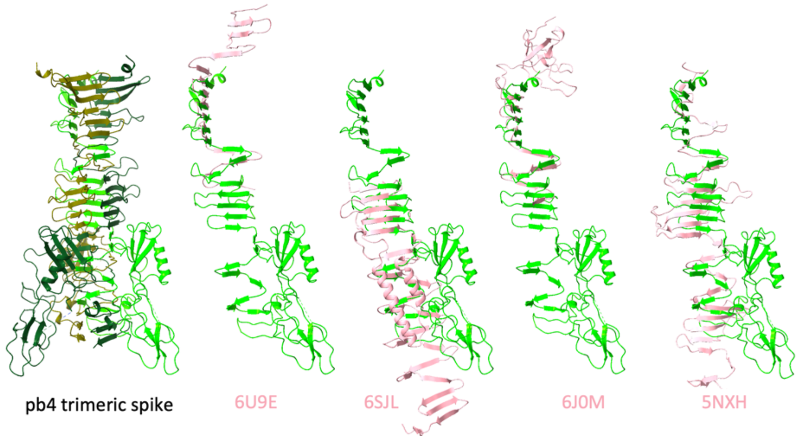

C

Protein name	Theoretical mass (Da) full/after processing	Observed mass (Da)	Processing
**STF$_{pb1}$**	147,992 / 132,708	132,708	Loss of N-ter Met, cleaved after T1,263
**TMP$_{pb2}$**	131,496 / 121,681 (residues 2-1,127) and 9,702 (1,128-1,219)	121,683- 9,702	Loss of N-ter Met, cleaved after R1,127
**BHP$_{pb3}$**	107,166	107,1984	-
**TTP$_{pb6}$**	50,414 / 50,282	50,283	Loss of N-ter Met
**DTP$_{pb9}$**	22,778	22,779	-
**p142**	18,360	18,361	-
**p143**	27,857 / 27,726	27,727	Loss of N-ter Met

Figure S8: Tip central fiber structure analysis (A,B) and MS analysis of T5 tails(C). (A) interface between BHP$_{pb3}$ last FNIII (yellow) and pb4 first FNIII (green) in ribbon representation. BHP$_{pb3}$ C-terminus (C) and pb4 N-terminus (N) are highlighted. Residues at the interface between the two proteins are shown in sticks. (**B**) Table of relevant DALI hits for pb4 spike (top panel), and alignment of a pb4 monomer with a monomer of the four first proteins of the table (bottom panel). (**C**) LC-ESI-TOF-MS of purified tails. The experimental intact mass of seven T5 tail proteins were determined. Even though proteomics detected their presence (Table S3), masses of pb4, RBP$_{pb5}$, p140 and p132 as intact proteins could not be measured. This may be explained by a difficulty in their ionisation.

Table S1: Cryo-EM data collection, refinement and validation statistics

	Common core (EMD-13953) (PDB 7QG9)	Tip (without pb4) (EMD-14733) (PDB 7ZHJ)	Tip (fibre) (EMD-14790) (PDB 7ZLV)	Tip (full) (EMD-14869) (PDB 7ZQB)
**Data collection and processing**				
Magnification	105.000 x	105.000 x	105.000 x	105.000 x
Voltage (kV)	300	300	300	300
Electron exposure (e^{-}/Å^2)	40	40	40	40
Defocus range (μm)	-1.0 to -3.0	-1.0 to -3.0	-1.0 to -3.0	-1.0 to -3.0
Pixel size (Å)	1.351	1.351	1.351	1.351
Symmetry imposed	C3	C3	C3	C3
Micrographs (no.)	12816	3208	3208	3208
Final particle images (no.)	29639	9290	9290	9290
Map resolution (Å) 0.143 FSC threshold	3.44	3.53	4.22	3.88
Map resolution range (Å)	3.2 - 4.5	3.3 - 5	4 - 6	3.5 - 7
**Refinement**				
Model resolution (Å) 0.5 FSC threshold	3.51	3.5	4.6	4
Map sharpening *B* factor (Å^2)	-112	-75	-123	-40
Model composition				
Chain count	27	33	6	36
Non-hydrogen atoms	50829	74346	12237	86583
Protein residues	6576	9528	1581	11109
Ligands	0	0	0	0
B factors (Å^2)				
Protein (min/max/mean)	17.76/180.35/61.99	17.76/180.5/55.05	83.25/292.09/136.25	17.76/292.09/66.53
Ligand	n/a	n/a	n/a	n/a
R.m.s. deviations				
Bond lengths (Å)	0.004	0.004	0.004	0.004
Bond angles (°)	0.955	0.945	0.993	0.952
Validation				
MolProbity score	1.69	1.7	2.46	1.87
Clashscore	8.97	8.47	25.02	10.81
Poor rotamers (%)	0	0.04	0	0.03
Ramachandran plot				
Favoured (%)	96.67	96.41	89.29	95.4
Allowed (%)	3.33	3.59	10.52	4.57
Disallowed (%)	0	0	0.19	0.03

	Tip-FhuA (full) (EMD-14799) (PDB 7ZN2)	Tip-FhuA (bent fibre) (EMD-14800) (PDB 7ZN4)	Tip-FhuA (open tube) (EMD-14873) (PDB 7ZQP)
**Data collection and processing**			
Magnification	105.000 x	105.000 x	105.000 x
Voltage (kV)	300	300	300
Electron exposure (e–/Å^2)	40	40	40
Defocus range (μm)	-1.0 to -3.0	-1.0 to -3.0	-1.0 to -3.0
Pixel size (Å)	1.351	1.351	1.351
Symmetry imposed	C1	C1	C3
Micrographs (no.)	9608	9608	9608
Final particle images (no.)	20349	20349	20349
Map resolution (Å) 0.143 FSC threshold	4.25	4.32	3.60
Map resolution range	4 - 20	4 - 6	3.4 - 4.5
**Refinement**			
Model resolution (Å) 0.5 FSC threshold	4.3	4.3	3.6
Map sharpening *B* factor (Å^2)	-90	-135	-141
Model composition			
Chain count	36	6	6
Non-hydrogen atoms	89393	19989	18315
Protein residues	11481	2581	2289
Ligands	0	0	0
B factors (Å^2)			
Protein (min/max/mean)	17.76/200.25/67.84	54.9/144.16/94.69	21.56/200.25/53.75
Ligand	n/a	n/a	n/a
R.m.s. deviations			
Bond lengths (Å)	0.004	0.004	0.004
Bond angles (°)	0.946	0.934	0.931
Validation			
MolProbity score	1.66	1.62	1.52
Clashscore	7.87	6.89	5
Poor rotamers (%)	0	0	0
Ramachandran plot			
Favoured (%)	96.49	96.36	96.18
Allowed (%)	3.51	3.64	3.82
Disallowed (%)	0	0	0

Table S2: **Domain definition of T5 tip proteins**. STF_{pb1}, RBP_{pb5} and p143, in italics, are partially or not resolved in our EM maps.

T5 protein name / function	Number of residues	Domains	Sequence	Colour
TPP_{pb6}<br>Tail Tube Protein	464	Tail tube domain1<br>Tail tube domain 2<br>Ig-Like	1-203<br>204-355<br>356-464	cyan
p140<br>Baseplate Tube Protein	298	Tail tube domain 1<br>Tail tube domain 2	1-167<br>168-298	blue
p132<br>Collar protein	140	Ig-like	1-140	purple
STF_{pb1}<br>*Side Tail Fibre*	*1,263*			pink
DTP_{pb9}<br>Distal Tail Protein	204	Tail tube domain<br>OB-Fold	1-83; 172-204<br>84-171	orange
BHP_{pb3}<br>Baseplate Hub Protein	949	hdI<br>hdII-Insertion<br>plug/β-hairpin<br>hdII<br>hdIII<br>hdIV<br>hdIV-FNIII linker<br>FNIII-1<br>FNIII-2	1-159<br>160-437<br>210-262<br>438-570<br>586-658<br>571-585; 659-709<br>711-742<br>743-835<br>836-949	gold
TMP_{pb2}<br>Tape Measure Protein	1,219	TMP_{pb2*}<br>TMP_{pb2C}	2-1127,<br>1128-1219	red
pb4<br>Central Fibre Protein	688	FNIII-1<br>FNIII-2<br>FNIII-3<br>FNIII-spike linker<br>Spike<br>Small domains	1-105<br>106-208<br>209-316<br>317-332<br>333-465; 627-688<br>466-626	lime
RBP_{pb5}<br>*Receptor Binding Protein*	*640*			dark green
p143<br>*Tail Completion Protein*	*262*			cornflower blue

Table S3: Proteomics of T5 tail proteins.

Protein	Accession number	Gene name	Protein set score	Theoretical mass (Da) of full protein	#observable peptides	Coverage %
**STF_{pb1}**	**FIBL1_BPT5**	**ltf**	**8529.02**	**147992**	**81**	**73.14**
**TMP_{pb2}**	**TMP_BPT5**	**D18-19**	**7609.99**	**131496**	**75**	**62.84**
**BHP_{pb3}**	**BPPB3_BPT5**	**D16**	**4554.46**	**107166**	**53**	**75.66**
**pb4**	**FIBC_BPT5**	**D17**	**3347.97**	**74788**	**31**	**68.02**
**RBP_{pb5}**	**RBP5_BPT5**	**oad**	**2063.73**	**68726**	**25**	**54.38**
**TTP_{pb6}**	**TUBE_BPT5**	**N4**	**5054.41**	**50414**	**22**	**95.69**
**p143**	**COMPL_BPT5**	**ORF136**	**970.79**	**27857**	**18**	**63.53**
**p140**	**TAIL1_BPT5**	**ORF133**	**1221.08**	**34334**	**12**	**62.75**
**DTP_{pb9}**	**DIT_BPT5**	**D16**	**1315.54**	**22778**	**11**	**85.29**
**p132**	**FIBL2_BPT5**	**ORF125**	**375.13**	**15067**	**5**	**57.86**
**p142**	**TTTP_BPT5**	**ORF135**	**650.65**	**18360**	**5**	**40.37**
MCP_{pb8}	CAPSD_BPT5	D20	537.11	50885	31	24.45
pb10	DECO_BPT5	N5	84	17247	11	9.15
putative metallopeptidase/ ribonuclease	Q5DML2_BPT5	ORF082	66.64	25001	12	10.36
Uncharacterized protein	Q66M03_BPT5	T5p046	256.58	27425	16	26.29
Glucose-1-phosphatase	AGP_ECOLI	agp	114.46	45683	24	6.05
Elongation factor Tu	EFTU1_ECOLI	tufA	86.7	43284	26	4.31
Uncharacterized protein	YAGL_ECOLI	yagL	29.97	27274	16	3.88
Glyceraldehyde-3-phosphate dehydrogenase A	G3P1_ECOLI	gapA	27.76	35532	24	3.32
Uncharacterized protein	YDHW_ECOLI	ydhW	26.29	24421	13	4.19

Bold proteins are tail proteins (*17*). MCP_{pb8} and pb10 are capsid proteins, identified because of a small proportion of revertant (10^{-7}) in the T5D20*am*30d mutant, leading to the production of full T5 particles.
Q66M03_BPT5: uncharacterised T5 protein, gene located in the early gene region. It is surrounded by genes coding for lysins, holins and endolysins.
Q5DML2_BPT5: T5 protein, annotated as a putative metallopeptidase/carboxypeptidase. HHPred aligns it with nucleases or a subunit of DNA polymerase III. It is located in the early gene portion of the genome, surrounded by DNA and RNA interacting proteins. Phagonaute (*58*) aligns it with similar proteins in T5-like phages, but also other sipho- and myo-phages.

Table S4: Validation statistics and model building for T5 tip individual proteins

Tip / Tip-FhuA common core proteins

**Protein**<br>Chain ID	**TTP_{pb6}-1**<br>D/E/F	**TTP_{pb6}-2**<br>A/B/C	**p132-1**<br>F/M/Q	**p132-2**<br>J/N/R	**p132-3**<br>K/O/S	**p132-4**<br>L/P/T	**p140**<br>G/H/I	**DTP_{pb9}-1**<br>V/X/Z	**DTP_{pb9}-2**<br>W/Y/a
Map(s) used for building	Tip / Tip-FhuA common core								
EMDB entry	EMD-13953								
Bond outliers (%)									
Lengths	0	0	0	0	0	0	0	0	0
Angles	0	0	0	0	0.07	0	0	0	0
MolProbity score	1.63	4.77	1.56	1.56	1.68	1.83	1.47	1.31	1.4
Clashscore	7.37	10.07	7.19	7.19	8.07	9.01	4.82	5.63	5.63
Rotamer outliers (%)	0	0	0	0	0	0	0	0	0
Ramachandran plot (%)									
Outliers	0	0	0	0	0	0	0	0	0
Allowed	3.46	3.68	2.92	2.92	3.62	5.07	3.38	1.98	2.48
Favored	96.54	96.32	97.08	97.08	96.38	94.93	96.62	98.02	97.52
Residues built/total	464/464	464/464	139/140	139/140	140/140	140/140	298/298	204/204	204/204

Tip proteins

**Protein**<br>Chain ID	**BHP_{pb3}**<br>b/c/d	**TMP_{pb2C}**<br>e/f/g	**pb4**<br>h/i/j
Map(s) used for building	Tip (without fibre)		First built into Tip-FhuA (bent fibre), then fitted and refined into Tip (fibre)
EMDB entry	EMD-14733		EMD-14790
Bond outliers (%)			
Lengths	0 (0)	0 (0)	0 (0)
Angles	0.01 (1)	0 (0)	0 (0)
MolProbity score	1.67	1.36	2.1
Clashscore	6.81	3.96	10.36
Rotamer outliers (%)	0.12	0	0
Ramachandran plot (%)			
Outliers	0	0	0.19
Allowed	4.22	3.03	10.52
Favored	95.78	96.97	89.29
Residues built/total	949/949	35/1219	527/688

Tip-FhuA proteins

**Protein**<br>Chain ID	**BHP_{pb3}-1**<br>b	**BHP_{pb3}-2**<br>c	**BHP_{pb3}-3**<br>d	**pb4-1**<br>h	**pb4-2**<br>I	**pb4-3**<br>j	**TMP_{pb2*}**<br>e/f/g
Map(s) used for building	Tip-FhuA (open tube): residues 1-710<br>Tip-FhuA (full): hdIV-FNIII linker, residues 711-729<br>Tip-FhuA (bent fibre): FNIIIs, residues 730-949			Tip-FhuA (bent fibre)			Tip-FhuA (open tube)<br>Tip-FhuA (full)
EMDB entry	EMD-14799<br>EMD-14800<br>EMD-14873			EMD-14800			EMD-14799<br>EMD-14873
Bond outliers (%)							
Lengths	0	0	0	0	0	0	0
Angles	0	0	0	0	0	0	0
MolProbity score	1.56	1.48	1.62	1.58	1.38	1.38	1.23
Clashscore	4.6	5.61	5.01	5.19	5.89	5	4.62
Rotamer outliers (%)	0	0	0	0	0	0	0
Ramachandran plot (%)							
Outliers	0	0	0	0	0	0	0
Allowed	4.65	3.06	5.17	4.36	2.26	90.57	0
Favored	95.35	96.94	94.83	95.64	97.74	9.43	100
Residues built/total	949/949	949/949	949/949	671/688	669/688	589/688	43/1219

Supplementary Movie S1 and S2| Morphs of the BHP_{pb3} trimer between the conformation before and after interaction with the receptor. Side (S1) and top (S2) views. Monomers are coloured yellow, orange and salmon, with the hdIV-FNIII linker and the two FNIII domains in darker shades of the same colour.

Supplementary Movie S3 and S4 | Morphs of the BHP_{pb3} trimer without its FNIIIs, between the conformation before and after interaction with the receptor. Side (S3) and top (S4) views. Monomers are coloured yellow, orange and salmon, with the hdI and hdIV domains in lighter colours, the hdIV-FNIII linker in dark orange and the plugs in brown.

REFERENCES AND NOTES

1. C. A. Suttle, Marine viruses--major players in the global ecosystem. *Nat. Rev. Microbiol.* **5**, 801–812 (2007).

2. S. Uyttebroek, B. Chen, J. Onsea, F. Ruythooren, Y. Debaveye, D. Devolder, I. Spriet, M. Depypere, J. Wagemans, R. Lavigne, J.-P. Pirnay, M. Merabishvili, P. De Munter, W. E. Peetermans, L. Dupont, L. Van Gerven, W.-J. Metsemakers, Safety and efficacy of phage therapy in difficult-to-treat infections: A systematic review. *Lancet Infect. Dis.* **22**, e208–e220 (2022).

3. D. Veesler, C. Cambillau, A common evolutionary origin for tailed-bacteriophage functional modules and bacterial machineries. *Microbiol. Mol. Biol. Rev.* **75**, 423–433 (2011).

4. A. R. Davidson, L. Cardarelli, L. G. Pell, D. R. Radford, K. L. Maxwell, Long noncontractile tail machines of bacteriophages. *Adv. Exp. Med. Biol.* **726**, 115–142 (2012).

5. M. Brackmann, S. Nazarov, J. Wang, M. Basler, Using force to punch holes: Mechanics of contractile nanomachines. *Trends Cell Biol.* **27**, 623–632 (2017).

6. R. Linares, C.-A. Arnaud, S. Degroux, G. Schoehn, C. Breyton, Structure, function and assembly of the long, flexible tail of siphophages. *Curr. Opin. Virol.* **45**, 34–42 (2020).

7. A. Desfosses, H. Venugopal, T. Joshi, J. Felix, M. Jessop, H. Jeong, J. Hyun, J. B. Heymann, M. R. H. Hurst, I. Gutsche, A. K. Mitra, Atomic structures of an entire contractile injection system in both the extended and contracted states. *Nat. Microbiol.* **4**, 1885–1894 (2019).

8. F. Jiang, N. Li, X. Wang, J. Cheng, Y. Huang, Y. Yang, J. Yang, B. Cai, Y.-P. Wang, Q. Jin, N. Gao, Cryo-EM structure and assembly of an extracellular contractile injection system. *Cell* **177**, 370–383.e15 (2019).

9. P. Ge, D. Scholl, N. S. Prokhorov, J. Avaylon, M. M. Shneider, C. Browning, S. A. Buth, M. Plattner, U. Chakraborty, K. Ding, P. G. Leiman, J. F. Miller, Z. H. Zhou, Action of a minimal contractile bactericidal nanomachine. *Nature* **580**, 658–662 (2020).

10. H.-W. Ackermann, Phage classification and characterization. *Methods Mol. Biol.* **501**, 127–140 (2009).

11. C.-A. Arnaud, G. Effantin, C. Vivès, S. Engilberge, M. Bacia, P. Boulanger, E. Girard, G. Schoehn, C. Breyton, Bacteriophage T5 tail tube structure suggests a trigger mechanism for *Siphoviridae* DNA ejection. *Nat. Commun.* **8**, 1953 (2017).

12. P. L. Campbell, R. L. Duda, J. Nassur, J. F. Conway, A. Huet, Mobile loops and electrostatic interactions maintain the flexible tail tube of bacteriophage lambda. *J. Mol. Biol.* **432**, 384–395 (2020).

13. A. Goulet, J. Lai-Kee-Him, D. Veesler, I. Auzat, G. Robin, D. A. Shepherd, A. E. Ashcroft, E. Richard, J. Lichière, P. Tavares, C. Cambillau, P. Bron, The opening of the SPP1 bacteriophage tail, a prevalent mechanism in gram-positive-infecting Siphophages. *J. Biol. Chem.* **286**, 25397–25405 (2011).

14. D. Veesler, S. Spinelli, J. Mahony, J. Lichière, S. Blangy, G. Bricogne, P. Legrand, M. Ortiz-Lombardia, V. Campanacci, D. van Sinderen, C. Cambillau, Structure of the phage TP901-1 1.8 MDa baseplate suggests an alternative host adhesion mechanism. *Proc. Natl. Acad. Sci. U.S.A.* **109**, 8954–8958 (2012).

15. G. Sciara, C. Bebeacua, P. Bron, D. Tremblay, M. Ortiz-Lombardia, J. Lichière, M. van Heel, V. Campanacci, S. Moineau, C. Cambillau, Structure of lactococcal phage p2 baseplate and its mechanism of activation. *Proc. Natl. Acad. Sci. U.S.A.* **107**, 6852–6857 (2010).

16. J. L. Kizziah, K. A. Manning, A. D. Dearborn, T. Dokland, Structure of the host cell recognition and penetration machinery of a *Staphylococcus aureus* bacteriophage. *PLOS Pathog.* **16**, e1008314 (2020).

17. Y. Zivanovic, F. Confalonieri, L. Ponchon, R. Lurz, M. Chami, A. Flayhan, M. Renouard, A. Huet, P. Decottignies, A. R. Davidson, C. Breyton, P. Boulanger, Insights into bacteriophage T5 structure from analysis of its morphogenesis genes and protein components. *J. Virol.* **88**, 1162–1174 (2014).

18. M. Demerec, U. Fano, Bacteriophage-resistant mutants in Escherichia coli. *Genetics* **30**, 119–136 (1945).

19. A. Huet, R. L. Duda, P. Boulanger, J. F. Conway, Capsid expansion of bacteriophage T5 revealed by high resolution cryoelectron microscopy. *Proc. Natl. Acad. Sci. U.S.A.* **116**, 21037–21046 (2019).

20. P. Boulanger, P. Jacquot, L. Plançon, M. Chami, A. Engel, C. Parquet, C. Herbeuval, L. Letellier, Phage T5 straight tail fiber is a multifunctional protein acting as a tape measure and carrying fusogenic and muralytic activities. *J. Biol. Chem.* **283**, 13556–13564 (2008).

21. C. Garcia-Doval, J. R. Castón, D. Luque, M. Granell, J. M. Otero, A. L. Llamas-Saiz, M. Renouard, P. Boulanger, M. J. van Raaij, Structure of the receptor-binding carboxy-terminal domain of the bacteriophage T5 L-shaped tail fiber with and without its intra-molecular chaperone. *Viruses* **7**, 6424–6440 (2015).

22. A. Flayhan, F. M. D. Vellieux, R. Lurz, O. Maury, C. Contreras-Martel, E. Girard, P. Boulanger, C. Breyton, Crystal Structure of pb9, the Distal tail protein of bacteriophage T5: A conserved structural motif among all siphophages. *J. Virol.* **88**, 820–828 (2014).

23. A. Flayhan, F. Wien, M. Paternostre, P. Boulanger, C. Breyton, New insights into pb5, the receptor binding protein of bacteriophage T5, and its interaction with its *Escherichia coli* receptor FhuA. *Biochimie* **94**, 1982–1989 (2012).

24. C. Breyton, A. Flayhan, F. Gabel, M. Lethier, G. Durand, P. Boulanger, M. Chami, C. Ebel, Assessing the conformational changes of pb5, the receptor-binding protein of phage T5, upon binding to its *Escherichia coli* receptor FhuA. *J. Biol. Chem.* **288**, 30763–30772 (2013).

25. V. Braun, FhuA (TonA), the career of a protein. *J. Bacteriol.* **191**, 3431–3436 (2009).

26. M. Bonhivers, A. Ghazi, P. Boulanger, L. Letellier, FhuA, a transporter of the Escherichia coli outer membrane, is converted into a channel upon binding of bacteriophage T5. *EMBO J.* **15**, 1850–1856 (1996).

27. N. Chiaruttini, M. de Frutos, E. Augarde, P. Boulanger, L. Letellier, V. Viasnoff, Is the in vitro ejection of bacteriophage DNA quasistatic? A bulk to single virus study. *Biophys. J.* **99**, 447–455 (2010).

28. I. G. Denisov, S. G. Sligar, Nanodiscs in membrane biochemistry and biophysics. *Chem. Rev.* **117**, 4669–4713 (2017).

29. S. Degroux, G. Effantin, R. Linares, G. Schoehn, C. Breyton, Deciphering bacteriophage T5 host recognition mechanism and infection trigger. *J. Virol.* **2023**, e01584–22 (2023).

30. H. Fraga, C.-A. Arnaud, D. F. Gauto, M. Audin, V. Kurauskas, P. Macek, C. Krichel, J.-Y. Guan, J. Boisbouvier, R. Sprangers, C. Breyton, P. Schanda, Solid-state NMR H-N-(C)-H and H-N-C-C 3D/4D correlation experiments for resonance assignment of large proteins. *ChemPhysChem* **18**, 2697–2703 (2017).

31. L. Holm, DALI and the persistence of protein shape. *Protein Sci.* **29**, 128–140 (2020).

32. N. M. I. Taylor, N. S. Prokhorov, R. C. Guerrero-Ferreira, M. M. Shneider, C. Browning, K. N. Goldie, H. Stahlberg, P. G. Leiman, Structure of the T4 baseplate and its function in triggering sheath contraction. *Nature* **533**, 346–352 (2016).

33. E. Krissinel, K. Henrick, Inference of macromolecular assemblies from crystalline state. *J. Mol. Biol.* **372**, 774–797 (2007).

34. M. Noirclerc-Savoye, A. Flayhan, C. Pereira, B. Gallet, P. Gans, C. Ebel, C. Breyton, Tail proteins of phage T5: Investigation of the effect of the His6-tag position, from expression to crystallisation. *Protein Expr. Purif.* **109**, 70–78 (2015).

35. J. Jumper, R. Evans, A. Pritzel, T. Green, M. Figurnov, O. Ronneberger, K. Tunyasuvunakool, R. Bates, A. Žídek, A. Potapenko, A. Bridgland, C. Meyer, S. A. A. Kohl, A. J. Ballard, A. Cowie, B. Romera-Paredes, S. Nikolov, R. Jain, J. Adler, T. Back, S. Petersen, D. Reiman, E. Clancy, M. Zielinski, M. Steinegger, M. Pacholska, T. Berghammer, S. Bodenstein, D. Silver, O. Vinyals, A. W. Senior, K. Kavukcuoglu, P. Kohli, D. Hassabis, Highly accurate protein structure prediction with AlphaFold. *Nature* **596**, 583–589 (2021).

36. M. Zweig, D. J. Cummings, Cleavage of head and tail proteins during bacteriophage T5 assembly: Selective host involvement in the cleavage of a tail protein. *J. Mol. Biol.* **80**, 505–518 (1973).

37. L. C. Tsui, R. W. Hendrix, Proteolytic processing of phage lambda tail protein gpH: Timing of the cleavage. *Virology* **125**, 257–264 (1983).

38. S. Kanamaru, P. G. Leiman, V. A. Kostyuchenko, P. R. Chipman, V. V. Mesyanzhinov, F. Arisaka, M. G. Rossmann, Structure of the cell-puncturing device of bacteriophage T4. *Nature* **415**, 553–557 (2002).

39. S. R. Casjens, R. W. Hendrix, Locations and amounts of major structural proteins in bacteriophage lambda. *J. Mol. Biol.* **88**, 535–545 (1974).

40. L. Cardarelli, L. G. Pell, P. Neudecker, N. Pirani, A. Liu, L. A. Baker, J. L. Rubinstein, K. L. Maxwell, A. R. Davidson, Phages have adapted the same protein fold to fulfill multiple functions in virion assembly. *Proc. Natl. Acad. Sci. U.S.A.* **107**, 14384–14389 (2010).

41. P. Bárdy, T. Füzik, D. Hrebík, R. Pantůček, J. Thomas Beatty, P. Plevka, Structure and mechanism of DNA delivery of a gene transfer agent. *Nat. Commun.* **11**, 3034 (2020).

42. L. T. Alexander, R. Lepore, A. Kryshtafovych, A. Adamopoulos, M. Alahuhta, A. M. Arvin, Y. J. Bomble, B. Böttcher, C. Breyton, V. Chiarini, N. B. Chinnam, W. Chiu, K. Fidelis, R. Grinter, G. D. Gupta, M. D. Hartmann, C. S. Hayes, T. Heidebrecht, A. Ilari, A. Joachimiak, Y. Kim, R. Linares, A. L. Lovering, V. V. Lunin, A. N. Lupas, C. Makbul, K. Michalska, J. Moult, P. K. Mukherjee, W. S. Nutt, S. L. Oliver, A. Perrakis, L. Stols, J. A. Tainer, M. Topf, S. E. Tsutakawa, M. Valdivia-Delgado, T. Schwede, Target highlights in CASP14: Analysis of models by structure providers. *Proteins* **89**, 1647–1672 (2021).

43. G. Guihard, P. Boulanger, L. Letellier, Involvement of phage T5 tail proteins and contact sites between the outer and inner membrane of Escherichia coli in phage T5 DNA injection. *J. Biol. Chem.* **267**, 3173–3178 (1992).

44. M. Zweig, D. J. Cummings, Structural proteins of bacteriophage T5. *Virology* **51**, 443–453 (1973).

45. E. Kandiah, T. Giraud, A. de Maria Antolinos, F. Dobias, G. Effantin, D. Flot, M. Hons, G. Schoehn, J. Susini, O. Svensson, G. A. Leonard, C. Mueller-Dieckmann, CM01: A facility for cryo-electron microscopy at the European Synchrotron. *Acta Crystallogr. D Struct. Biol.* **75**, 528–535 (2019).

46. X. Li, P. Mooney, S. Zheng, C. R. Booth, M. B. Braunfeld, S. Gubbens, D. A. Agard, Y. Cheng, Electron counting and beam-induced motion correction enable near-atomic-resolution single-particle cryo-EM. *Nat. Methods* **10**, 584–590 (2013).

47. K. Zhang, Gctf: Real-time CTF determination and correction. *J. Struct. Biol.* **193**, 1–12 (2016).

48. G. Tang, L. Peng, P. R. Baldwin, D. S. Mann, W. Jiang, I. Rees, S. J. Ludtke, EMAN2: An extensible image processing suite for electron microscopy. *J. Struct. Biol.* **157**, 38–46 (2007).

49. J. Zivanov, T. Nakane, B. O. Forsberg, D. Kimanius, W. J. Hagen, E. Lindahl, S. H. Scheres, New tools for automated high-resolution cryo-EM structure determination in RELION-3. *eLife* **7**, e42166 (2018).

50. C.-A. Arnaud, thesis, University Grenoble-Alpes (2017).

51. P. Emsley, B. Lohkamp, W. G. Scott, K. Cowtan, Features and development of Coot. *Acta Crystallogr. D Biol. Crystallogr.* **66**, 486–501 (2010).

52. P. D. Adams, P. V. Afonine, G. Bunkóczi, V. B. Chen, I. W. Davis, N. Echols, J. J. Headd, L.-W. Hung, G. J. Kapral, R. W. Grosse-Kunstleve, A. J. McCoy, N. W. Moriarty, R. Oeffner, R. J. Read, D. C. Richardson, J. S. Richardson, T. C. Terwilliger, P. H. Zwart, PHENIX: A comprehensive Python-based system for macromolecular structure solution. *Acta Crystallogr. D Biol. Crystallogr.* **66**, 213–221 (2010).

53. C. J. Williams, J. J. Headd, N. W. Moriarty, M. G. Prisant, L. L. Videau, L. N. Deis, V. Verma, D. A. Keedy, B. J. Hintze, V. B. Chen, S. Jain, S. M. Lewis, W. B. Arendall III, J. Snoeyink, P. D. Adams, S. C. Lovell, J. S. Richardson, D. C. Richardson, MolProbity: More and better reference data for improved all-atom structure validation. *Protein Sci.* **27**, 293–315 (2018).

54. A. P. Pandurangan, M. Topf, RIBFIND: A web server for identifying rigid bodies in protein structures and to aid flexible fitting into cryo EM maps. *Bioinformatics* **28**, 2391–2393 (2012).

55. R. T. Kidmose, J. Juhl, P. Nissen, T. Boesen, J. L. Karlsen, B. P. Pedersen, Namdinator - automatic molecular dynamics flexible fitting of structural models into cryo-EM and crystallography experimental maps. *IUCrJ* **6**, 526–531 (2019).

56. M. G. Casabona, Y. Vandenbrouck, I. Attree, Y. Couté, Proteomic characterization of Pseudomonas aeruginosa PAO1 inner membrane. *Proteomics* **13**, 2419–2423 (2013).

57. J. Cox, M. Mann, MaxQuant enables high peptide identification rates, individualized p.p.b.-range mass accuracies and proteome-wide protein quantification. *Nat. Biotechnol.* **26**, 1367–1372 (2008).

58. H. Delattre, O. Souiai, K. Fagoonee, R. Guerois, M.-A. Petit, Phagonaute: A web-based interface for phage synteny browsing and protein function prediction. *Virology* **496**, 42–50 (2016).
